# A binding site for phosphoinositides described by multiscale simulations explains their modulation of voltage-gated sodium channels

**DOI:** 10.7554/eLife.91218

**Published:** 2024-03-11

**Authors:** Yiechang Lin, Elaine Tao, James P Champion, Ben Corry

**Affiliations:** 1 Research School of Biology, Australian National University Canberra Australia; RMIT University Australia; Stanford University United States

**Keywords:** sodium channel, molecular dynamics, phosphoinositide, lipid-protein interaction, inactivation, ion channel, None

## Abstract

Voltage-gated sodium channels (Naᵥ) are membrane proteins which open to facilitate the inward flux of sodium ions into excitable cells. In response to stimuli, Naᵥ channels transition from the resting, closed state to an open, conductive state, before rapidly inactivating. Dysregulation of this functional cycle due to mutations causes diseases including epilepsy, pain conditions, and cardiac disorders, making Naᵥ channels a significant pharmacological target. Phosphoinositides are important lipid cofactors for ion channel function. The phosphoinositide PI(4,5)P_2_ decreases Naᵥ1.4 activity by increasing the difficulty of channel opening, accelerating fast inactivation and slowing recovery from fast inactivation. Using multiscale molecular dynamics simulations, we show that PI(4,5)P_2_ binds stably to inactivated Naᵥ at a conserved site within the DIV S4–S5 linker, which couples the voltage-sensing domain (VSD) to the pore. As the Naᵥ C-terminal domain is proposed to also bind here during recovery from inactivation, we hypothesize that PI(4,5)P_2_ prolongs inactivation by competitively binding to this site. In atomistic simulations, PI(4,5)P_2_ reduces the mobility of both the DIV S4–S5 linker and the DIII–IV linker, responsible for fast inactivation, slowing the conformational changes required for the channel to recover to the resting state. We further show that in a resting state Naᵥ model, phosphoinositides bind to VSD gating charges, which may anchor them and impede VSD activation. Our results provide a mechanism by which phosphoinositides alter the voltage dependence of activation and the rate of recovery from inactivation, an important step for the development of novel therapies to treat Naᵥ-related diseases.

## Introduction

Voltage-gated sodium (Naᵥ) channels are critical to the regulation of brain activity, cardiac rhythm, and muscle contraction. Expressed in the membranes of excitable cells, Naᵥ channels respond to membrane depolarization to open a pore that facilitates the selective flow of sodium current into the cell, initiating the action potential. In mammals, the Naᵥ channel family consists of nine subtypes (Naᵥ1.1–1.9), distributed throughout the central and peripheral nervous system, as well as in cardiac and skeletal muscle (Yu and Catterall, 2003). The Naᵥ1.4 subtype is predominantly expressed in skeletal myofibers, where it initiates muscle contraction. Genetic mutations in this subtype are associated with various motor dysfunctions, such as both hyperkalemic and hypokalemic periodic paralyses (Mantegazza et al., 2021; Venance et al., 2006; Jurkat-Rott et al., 2000). Naᵥ1.7 is found in peripheral sensory neurons and is responsible for nociception. Several pain disorders, such as inherited erythromelalgia and small fiber neuropathy arise from gain-of-function Naᵥ1.7 mutations (Dib-Hajj et al., 2013). Both Naᵥ subtypes have been investigated as promising pharmacological targets for the treatment of myopathy and pain conditions (Dib-Hajj et al., 2013; Ghovanloo et al., 2021).

**Figure 1.**
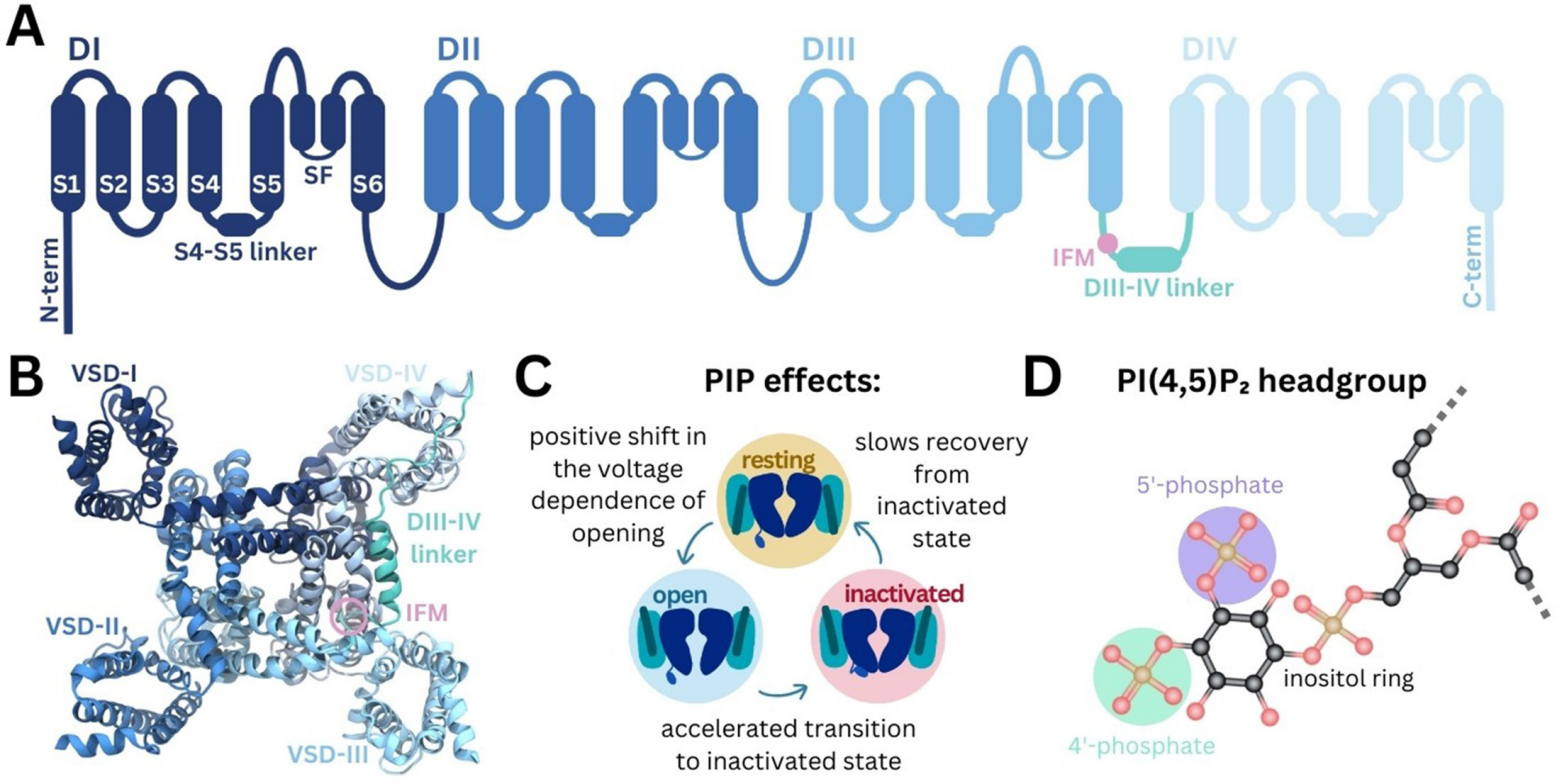
Structure of Naᵥ and modulation by phosphoinositides. (**A**) Naᵥ channel topology featuring transmembrane helices (S1–S6), the selectivity filter (SF), and the DIII–IV linker (containing the IFM motif) located between DIII and DIV. (**B**) Naᵥ1.4 structure (6agf) showing the four domain-swapped voltage-sensing domains (VSDs I–IV), pore, and DIII–IV linker on the intracellular side. (**C**) Summary of PI(4,5)P_2_ effects on transitions between Naᵥ channel functional states (Gada et al., 2023). (**D**) Structure of the PI(4,5)P_2_ headgroup with the 4’- and 5’-phosphates indicated.

Structurally, Naᵥ channels consist of four homologous domains (DI–DIV) arranged in a domain-swapped configuration (Figure 1A–B). Each domain comprises six transmembrane helices (S1–S6). The central pore domain is formed by S5, S6 helices and selectivity filter (SF), while the four peripheral VSDs are formed by S1–S4 (de Lera Ruiz and Kraus, 2015; Pan et al., 2018). The pore domain also features lateral fenestrations that provide a pathway for the access of small molecules to the pore via the membrane (Gamal El-Din et al., 2018) and have been shown in computational studies to be accessible to lipid tails (Raju et al., 2013; Boiteux et al., 2014; Martin and Corry, 2014; Tao and Corry, 2022). Additionally, the C-terminal domain (CTD) extends from the DIV S6 helix into the cytoplasm, where it is thought to associate with the DIII–IV and DIV S4–S5 linkers in the resting state (Clairfeuille et al., 2019).

Naᵥ channels adopt distinct functional states during the cycle of membrane depolarization and repolarization (Figure 1C). In the resting state, the pore is closed and VSD S4 helices are in the down, deactivated state. Membrane depolarization triggers the asynchronous transition of VSD I–III S4 helices to the up, activated conformation, causing Naᵥ channels to open (Cha et al., 1999; Capes et al., 2013; Goldschen-Ohm et al., 2013). During prolonged depolarization, VSD-IV moves upward, causing the channels to adopt a fast-inactivated state in which a three-residue Ile-Phe-Met (IFM) hydrophobic motif located on the intracellular linker between DIII and DIV (DIII–IV linker) allosterically closes the pore (Yan et al., 2017). The CTD is proposed to bind at the S4–S5 linker of VSD-IV and sequester the DIII-IV linker during the resting state. During fast inactivation, the CTD dissociates from VSD-IV and releases the DIII–IV linker to allow IFM binding (Clairfeuille et al., 2019). Upon repolarization, the VSDs deactivate, the IFM motif disassociates, the CTD rebinds to VSD-IV, and the pore returns to the resting state.

Phosphoinositides (PIPs) are important cellular signaling molecules found on the cytoplasmic leaflet of the mammalian cell membrane. They can exist in seven forms, with phosphorylation possible at one (PIP1), two (PIP2), or all three (PIP3) positions on the inositol ring, at the 3’, 4’, and/or 5’ carbons. PIPs, particularly PI(4,5)P_2_, featuring phosphates at the 4’ and 5’ carbon positions (Figure 1D), are known to bind and modulate the activity of numerous ion channel families (Hille et al., 2015). These include voltage-gated ion channels, some of which have been resolved with PI(4,5)P_2_ bound (Sun and MacKinnon, 2020; Gao et al., 2021). PIP is known to interact with the VSDs of different potassium channels, to stabilize the positive gating charges and support the voltage-sensing mechanism (Schmidt et al., 2006). By also binding at the VSD-pore interface in channels such as Kᵥ7.1, PIP is proposed to facilitate coupling of VSD movement to pore opening (Sun and MacKinnon, 2020; Kasimova et al., 2015; Ma et al., 2022). PI(4,5)P_2_ also forms specific interactions with VSD-II of Caᵥ2.2 in the down state, making channel activation more difficult (Gao et al., 2021). Although PI(4,5)P_2_ is known to bind to numerous voltage-gated ion channels, its effects on channel gating and function are complex and yet to be fully elucidated.

Recent experiments show that Naᵥ1.4 channel kinetics and voltage dependence are modulated by PI(4,5)P_2_ (Gada et al., 2023). PI(4,5)P_2_ inhibits Naᵥ1.4 by causing a depolarizing shift in voltage dependence of activation, accelerating transition to the inactivated state and slowing recovery from inactivation, resulting in reduced peak current and suppression of late current (summarized in Figure 1C). While this is likely to occur via a direct interaction with Naᵥ1.4, the structural basis of PI(4,5)P_2_ modulation remains to be understood.

Here, we used a combination of coarse-grained and atomistic MD simulations to identify a putative PIP binding site to inactivated Naᵥ1.4 in VSD-IV and the DIII–IV linker. We analyze the atomistic level interactions between the positively charged residues at this site with PI(4,5)P_2_ and PI(4)P, comparing this with structurally resolved PIP binding sites in the related ion channels Caᵥ2.2 and Kᵥ7.1. Consistent with the sequence conservation at the identified site, we find that PIPs also bind to Naᵥ1.7 in coarse-grained simulations, with notable differences dependent on VSD conformation states. This work provides insight into how PIPs can negatively regulate Naᵥ channels, a first step for the potential development of PIP-analogue sodium channel inhibitors.

## Results

**Figure 2.**
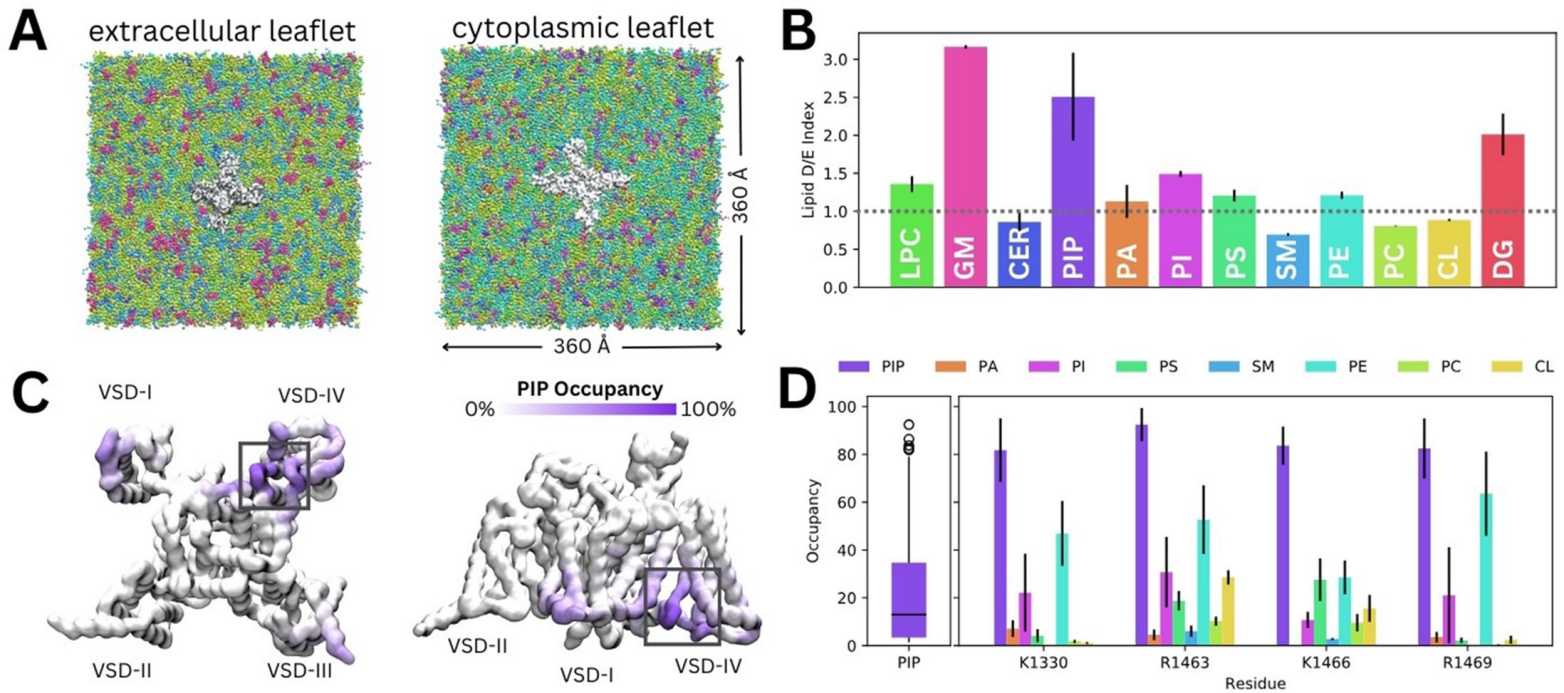
Lipid fingerprint and binding of all phosphoinositide (PIP) types to Naᵥ1.4. (**A**) Naᵥ1.4 embedded in a 360 Å × 360 Å model mammalian membrane containing 63 lipid species. (**B**) Lipid depletion enrichment index of lipids around Naᵥ1.4 grouped into 12 headgroup classes. (**C**) Naᵥ1.4, shown from the intracellular (left) and membrane (right) sides, colored by PIP occupancy (darker purple = greater PIP occupancy). (**D**) Distribution of PIP binding occupancies (left) and occupancy of lipid species at four residues with the highest PIP occupancy. Error bars show standard error, n = 3.

To investigate how diverse lipid species interact with Naᵥ1.4, we carried out coarse-grained simulations of Naᵥ1.4 (PDB ID: 6agf, inactivated state) embedded in a complex mammalian membrane for 16 µs in triplicate (Figure 2A). Glycosphingolipid, PIP, and diacylglycerol (DG) were highly enriched around Naᵥ1.4 (Figure 2B). Additionally, we observed modest enrichment of lysophosphatidylcholine (LPC), phosphatidylinositol (PI), phosphatidylserine (PS), and phosphatidylethanolamine, and slight depletion of ceramide, sphingomyelin, phosphatidylcholine, and cholesterol. To investigate specific interactions, we generated z-density maps and calculated the per-residue occupancy of the 12 different lipid types (Figure 2C, Figure 2—figure supplements 1–2). Binding residues of interest were identified by constructing occupancy distributions by residue for each lipid type and identifying outlying values with high occupancies (Figure 2—figure supplement 3). DG lipids form significant interactions within the lateral fenestrations of Naᵥ1.4 (Figure 2—figure supplement 2). LPC and PI also frequently interact with different VSD residues (Figure 2—figure supplement 1).

**Figure 2—figure supplement 1.**
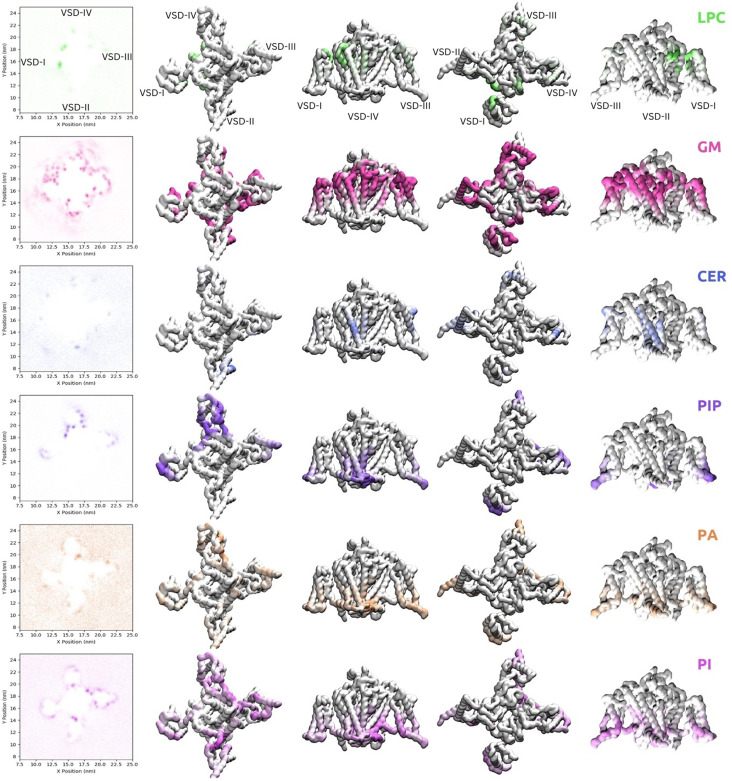
Lipid z-density maps (left) and contact occupancy structures (right) for all 12 lipid classes in the mammalian membrane. Shows lipid species (LPC), glycosphingolipid (GM), ceramide (CER), phosphoinositide (PIP), phosphatidic acid (PA), and phosphotidylinositol (PI). Density maps for each lipid type are show on the right. Contact occupancy structures are visualized from the intracellular view, the voltage-sensing domain (VSD)-IV side view, the extracellular view and the VSD-II side view (left to right).

**Figure 2—figure supplement 2.**
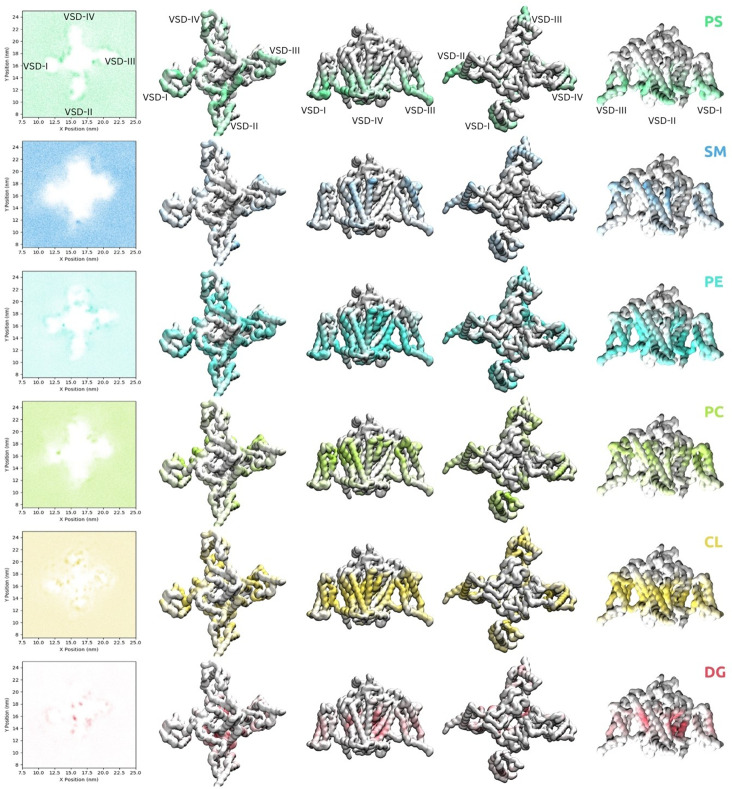
Lipid z-density maps (left) and contact occupancy structures (right) for all 12 lipid classes in the mammalian membrane. Shows phosphatidylserine (PS), sphingomyelin (SM), phosphatidylethanolamine (PE), phosphatidylcholine (PC), cholesterol (CL), and diacylglycerol (DG). Density maps for each lipid type are show on the right. Contact occupancy structures are visualized from the intracellular view, the voltage-sensing domain (VSD)-IV side view, the extracellular view and the VSD-II side view (left to right).

**Figure 2—figure supplement 3.**
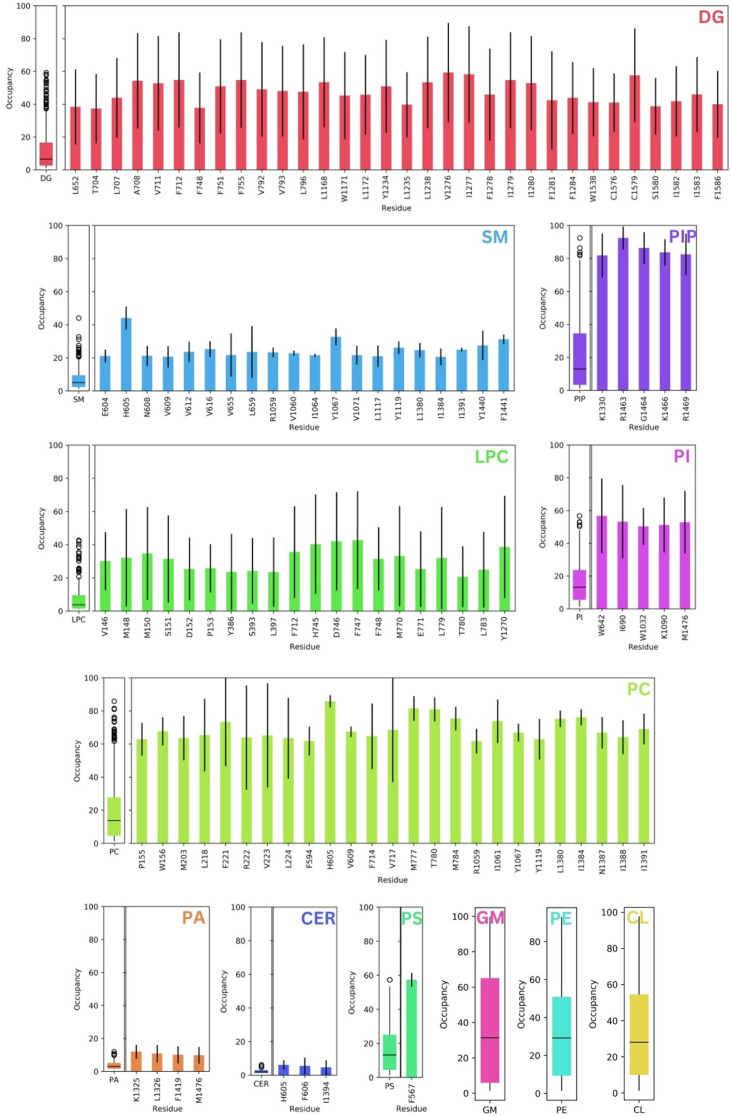
Contact occupancy distributions (left) and outlying residues (right) for all 12 lipid classes in the mammalian membrane. Error bars show standard error, n = 3.

Given our interest in understanding the modulation of Naᵥ1.4 by PIPs, we focus on their interactions for the remainder of this manuscript. Despite the very low concentration of PIPs (0.5% each of PIP1, PIP2, and PIP3) in the mammalian membrane, they are highly enriched around Naᵥ1.4, particularly near the DIII–IV linker and the VSD-IV (Figure 2C). Contact analysis revealed a putative PIP binding site involving the K1330 residue of the DIII–IV linker and residues R1463, K1466, and R1469 in the DIV S4–S5 linker, which connects the pore and VSD-IV (Figure 2D). Across all three replicates, only residues within this site were occupied by PIPs for more than 80% (quartile 3+1.5×interquartile range) of simulation time on average (Figure 2D). We note that these residues have higher occupancies for PIP compared to other lipids, including other negatively charged phospholipids (PA, PS, and PI) (Figure 2D).

**Figure 3.**
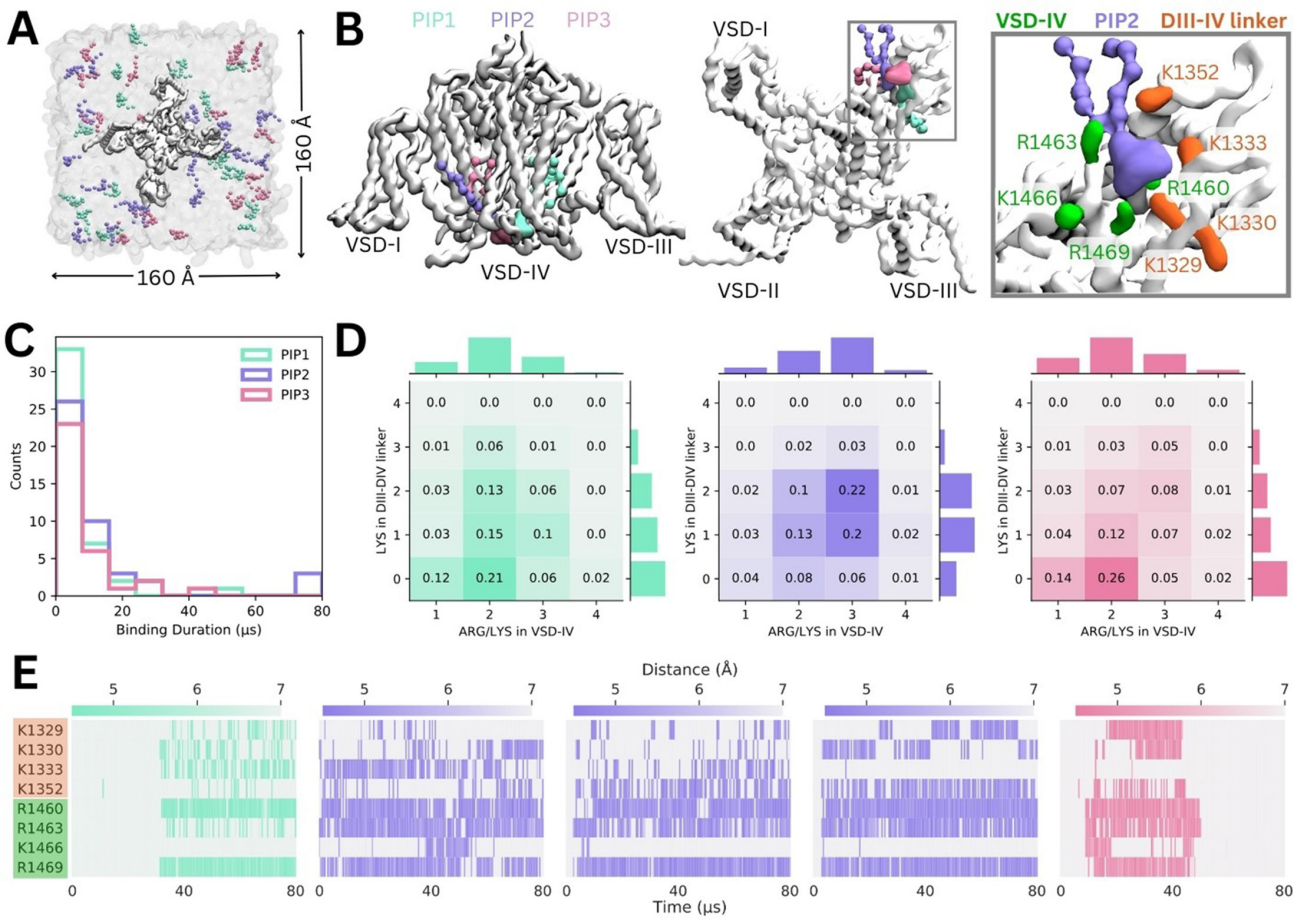
Binding of different phosphoinositide (PIP) species in enriched PIP simulations. (**A**) Enriched PIP simulation system, with Naᵥ1.4 embedded in a POPC membrane (transparent gray) and 5% each of PIP1 (blue), PIP2 (purple), and PIP3 (pink) added to the cytoplasmic leaflet. (**B**) Representative snapshots from the five longest binding events from different replicates, showing the three different PIP species (PIP1 in blue, PIP2 in purple, and PIP3 in pink) binding to voltage-sensing domain (VSD)-IV and the DIII–IV linker. Naᵥ1.4 is shown in white with interacting residues on the DIV S4–S5 linker and the DIII–IV linker colored in green and orange, respectively. (**C**) A frequency distribution showing interaction times for each PIP species, defined as the length of a continuous period in which a PIP was within 0.7 nm of two VSD-IV binding site residues. (**D**) Frequency plots showing number of positive residues interacting with bound PIP in the DIII–IV linker (vertical) and VSD-IV (horizontal). (**E**) Minimum distance between binding residues on Naᵥ1.4 and bound PIPs lipid across simulation time for the five longest binding events, colored by distance and the type of PIP bound.

Simulations in a complex mammalian membrane showed that PIP species bind specifically and selectively to Naᵥ1.4 in the presence of other negatively charged lipids and at low, physiological concentrations. However, the large membrane required, and long PIP binding durations prevented sampling of large numbers of binding and unbinding events. To address this, we carried out additional simulations where Naᵥ1.4 was placed in a smaller POPC membrane with a 5% concentration of each PIP species in the cytoplasmic leaflet (Figure 3A).

**Figure 3—figure supplement 1.**
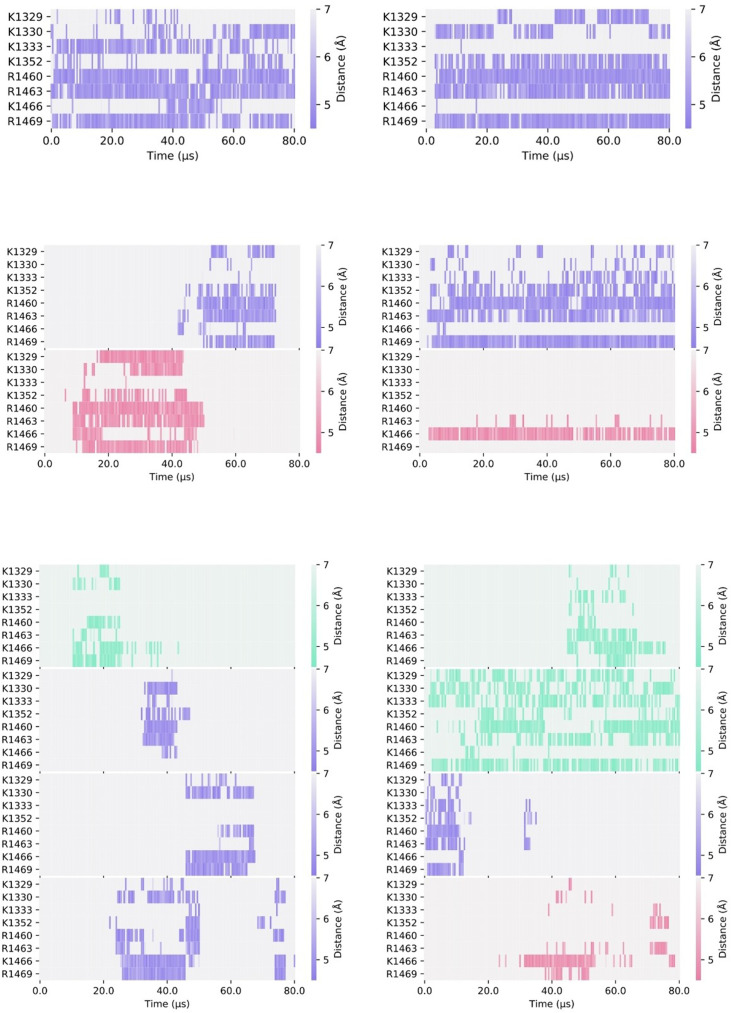
Minimum distance between binding residues on Naᵥ1.4 and bound phosphoinositides (PIPs) lipid across 31 long duration binding events (>10 µs), colored by distance and the type of PIP bound: PIP1 (blue-green), PIP2 (purple), and PIP3 (pink).

**Figure 3—figure supplement 2.**
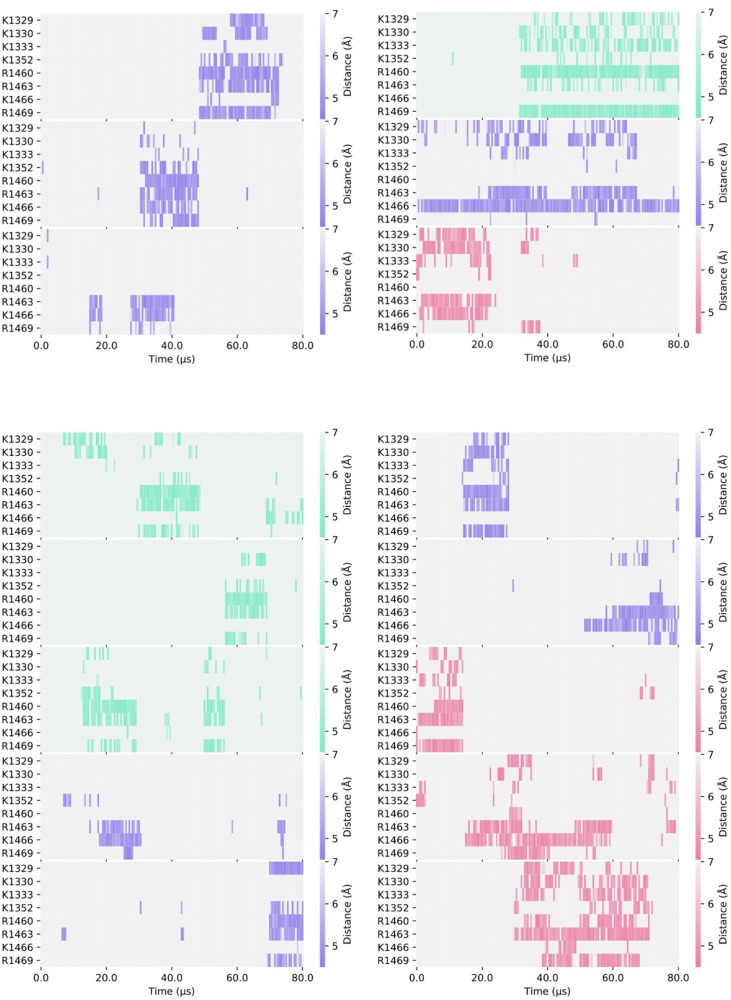
Minimum distance between binding residues on Naᵥ1.4 and bound phosphoinositides (PIPs) lipid across 31 long duration binding events (>10 µs), colored by distance and the type of PIP bound: PIP1 (blue-green), PIP2 (purple), and PIP3 (pink).

**Figure 3—figure supplement 3.**
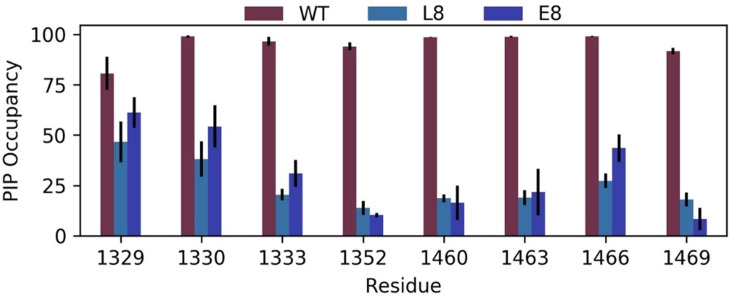
Phosphoinositide (PIP) occupancy at putative binding site residues in WT (wild-type) (brown) and when all eight binding site residues are mutated to leucine (L8, light blue) or glutamate (E8, dark blue). Error bars show standard error, n = 3.

Across 10 replicates of these enriched PIP simulations, each carried out for 80 µs, we observed all three PIP species binding at the identified site on VSD-IV and DIII–IV linker (Figure 3B). PIPs can approach and bind to this site from either side of the VSD, however, PIP1 only forms stable interactions when it approaches and binds from the VSD-I side while PIP2 and PIP3 usually bind from the VSD-III side (Figure 3B). These enriched PIP simulations also revealed additional positively charged residues in the DIII–IV linker (K1329, K1333, and K1352) and DIV S4 (R1460) which support binding.

There were 156 PIP binding/unbinding events with duration greater than 2 µs occurring in the identified site (Figure 3C). Of these, 43 were with PIP1, 44 with PIP2, and 33 with PIP3. The number of short-term (2–10 µs) interactions decreased with headgroup charge. That is, PIP1 formed the greatest number of short-term interactions while PIP3 had the fewest. Of the 31 binding events with duration greater than 10 µs, 7 were with PIP1, 15 with PIP2, and 9 with PIP3 (Figure 3—figure supplements 1–2).

When we analyzed interactions occurring during 2–80+ µs binding events, we found that the number of interacting basic residues changes depending on the PIP headgroup charge (Figure 3D). PIP1 (headgroup charge: –3e) binding is most frequently coordinated by two positive charges in VSD-IV and zero or one residue in the DIII–IV linker. PIP2 (headgroup charge: –5e) binding most frequently involves one or two interactions from the DIII–IV linker and three from VSD-IV for a total of five interactions. Interestingly, despite its greater negative charge, PIP3 (headgroup charge: –7e) interacts similarly to PIP1, and has fewer interactions than PIP2.

The minimum distance between the interacting PIP headgroup and each binding residue across simulation time is shown for the five 40+ µs PIP binding events (Figure 3E). Of these, three are PIP2 binding events which almost span the entire 80 µs of simulation. One 40+ µs binding event each for PIP1 and PIP3 were also observed. The stable PIP1 binding event observed involved interactions with R1460 and R1469 in VSD-IV, as well as fluctuating interactions with the four residues of the DIII–IV linker (K1329, K1330, K1333, K1352). The three long-term PIP2 binding events observed were similar to each other, mainly stabilized by R1460, R1463, and R1469. As with PIP1, the number and identity of interacting DIII–IV linker residues varied across the span of each simulation and between replicates, owing to the flexibility of the linker. Like PIP2, PIP3 binding was characterized by stable interactions with R1460, R1463, and R1469 (VSD-IV) as well as K1329 and K1330 (DIII–IV linker). In silico mutation of the eight residues implicated in PIP binding to leucine (charge neutralization while preserving side chain size) or glutamate (charge reversal) significantly reduced PIP binding (Figure 3—figure supplement 3).

Coarse-grained simulations enhance sampling by reducing the number of particles, enabling larger time steps and construction of a smoother energy surface. To examine interactions and protein conformational changes in atomistic detail, we backmapped representative snapshots from our PIP-enriched simulations, where we observed stable, long-term binding events between Naᵥ1.4 and PIP1/PIP2. The coarse-grained PIP1 and PIP2 molecules were converted to the atomistic PI(4)P and PI(4,5)P_2_, respectively. For each system, 7.5 µs of atomistic simulations were performed (5 replicates, 1.5 µs each).

**Figure 4.**
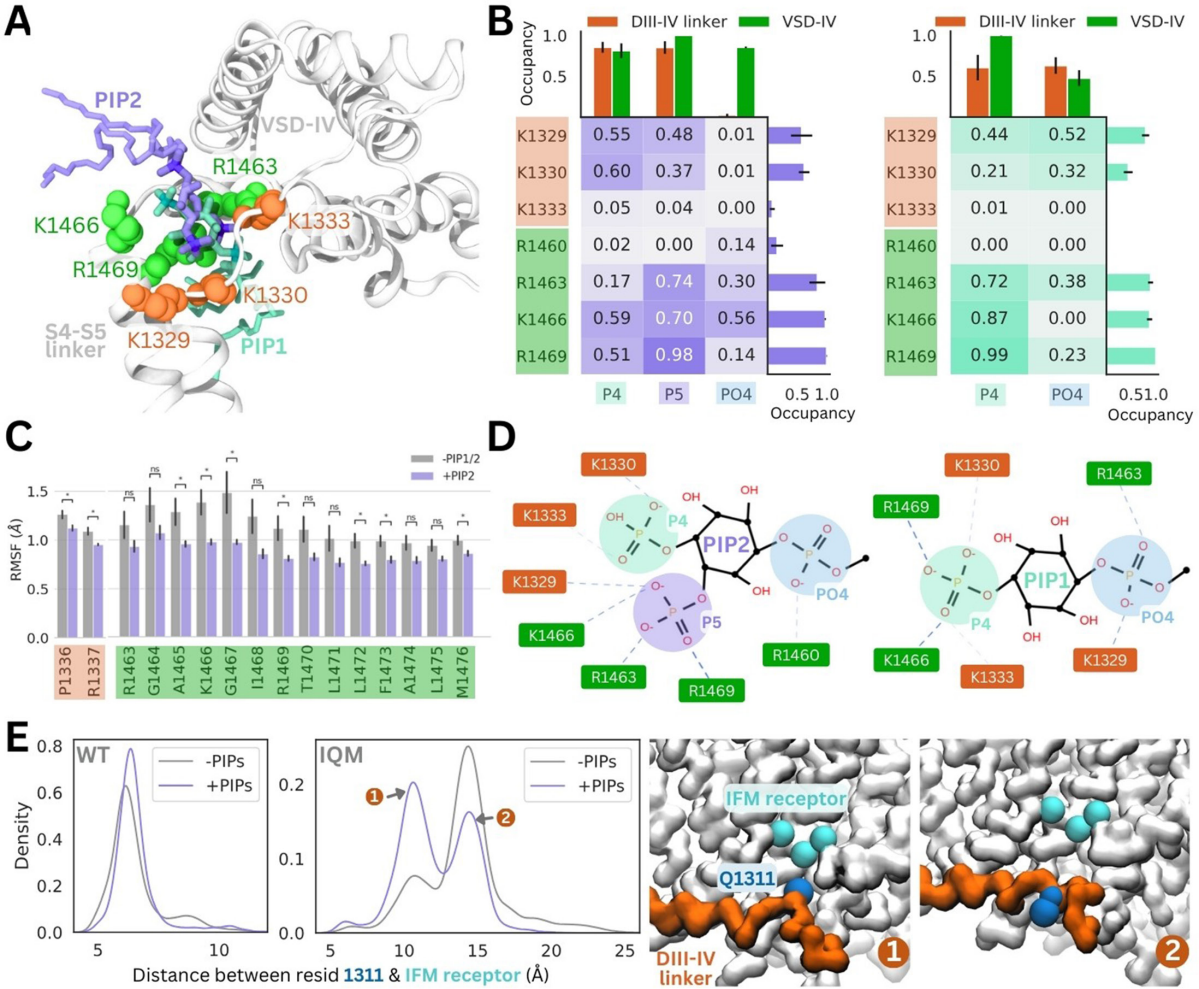
PI(4,5)P_2_ and PI(4)P binding to Naᵥ1.4 stabilizes the DIII–IV linker in atomistic and flexible coarse-grained simulations. (**A**) Representative snapshots of PI(4,5)P_2_ bound from the voltage-sensing domain (VSD)-I side (purple stick) and PI(4)P bound from the VSD-III side (cyan stick), with six basic residues forming the binding site located on the DIII–IV linker (orange VDW representation) and VSD-IV S4–S5 linker (shown in green VDW representation) visualized from the intracellular face of the protein. (**B**) Proportion of frames where each of the binding site residues were identified to be within 4.5 Å with the different headgroup regions, P4, P5, and PO4, for PI(4,5)P_2_ (left) and PI(4)P (right). Error bars show standard error, n = 5. (**C**) Comparison of RMSF per carbon-alpha for simulations with and without bound PI(4,5)P_2_, showing residues on the S4–S5 linker and DIV linker with significant differences in mobility (student’s t-test, p-value <0.05). (**D**) Interaction network plots between the phosphoinositide (PIP) headgroup and basic binding residues on DIII–IV linker (orange) and DIV S4–S5 linker (green), generated by ProLIF – showing the dominant interactions across simulations of PI(4,5)P_2_ and PI(4)P. (**E**) Density plots showing differences in the distributions of distance between IFM/IQM motif and its binding pocket in the presence and absence of PIPs for the Naᵥ1.4 wild-type (left) and IFM->IQM mutant (right); with representative snapshots showing the two distinct conformations of the IQM motif in the mutant.

In these simulations, the PI(4,5)P_2_ headgroup was stable at the binding site identified in coarse-grained simulations (root mean square deviation [RMSD] <2.2 Å) (Figure 4A, Figure 4—figure supplement 1). PI(4,5)P_2_ binding is predominantly coordinated by R1469 on the DIV S4–S5 linker, as well as R1466 and R1463. In one replicate, PI(4,5)P_2_ associates with R1460 via the phosphate group (PO4) that connects the headgroup to the PIP tails. The PI(4,5)P_2_ headgroup also forms electrostatic interactions with K1329 and K1330 in the DIII–IV linker, but forms few contacts with K1333 (Figure 4B, Figure 4—figure supplement 2). This loop portion of the DIII–IV linker is highly flexible (Figure 4—figure supplement 1), thus the lysine side chains can flip between binding the PI(4,5)P_2_ headgroup and facing the intracellular space.

**Figure 4—figure supplement 1.**
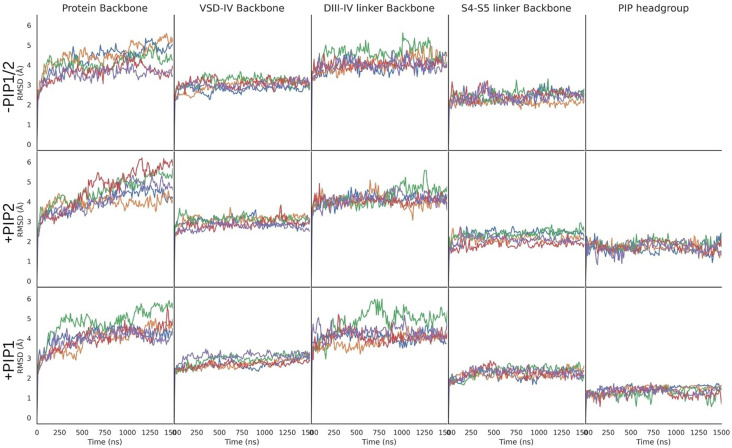
Root mean square deviation (RMSD) of the Naᵥ1.4 backbone, voltage-sensing domain (VSD)-IV backbone, DIII–IV linker backbone, and S4–S5 backbone (and PI(4,5)P_2_/PI(4)P_1_ headgroup) over 1.5 µs of atomistic simulations without any phosphoinositide (PIP) (top row), with a single PI(4,5)P_2_ lipid bound (middle row) and with PI(4)P_1_ bound (bottom row). Five replicates were simulated – rep1 (blue), rep2 (red), rep3 (green), rep4 (orange), rep5 (purple).

**Figure 4—figure supplement 2.**
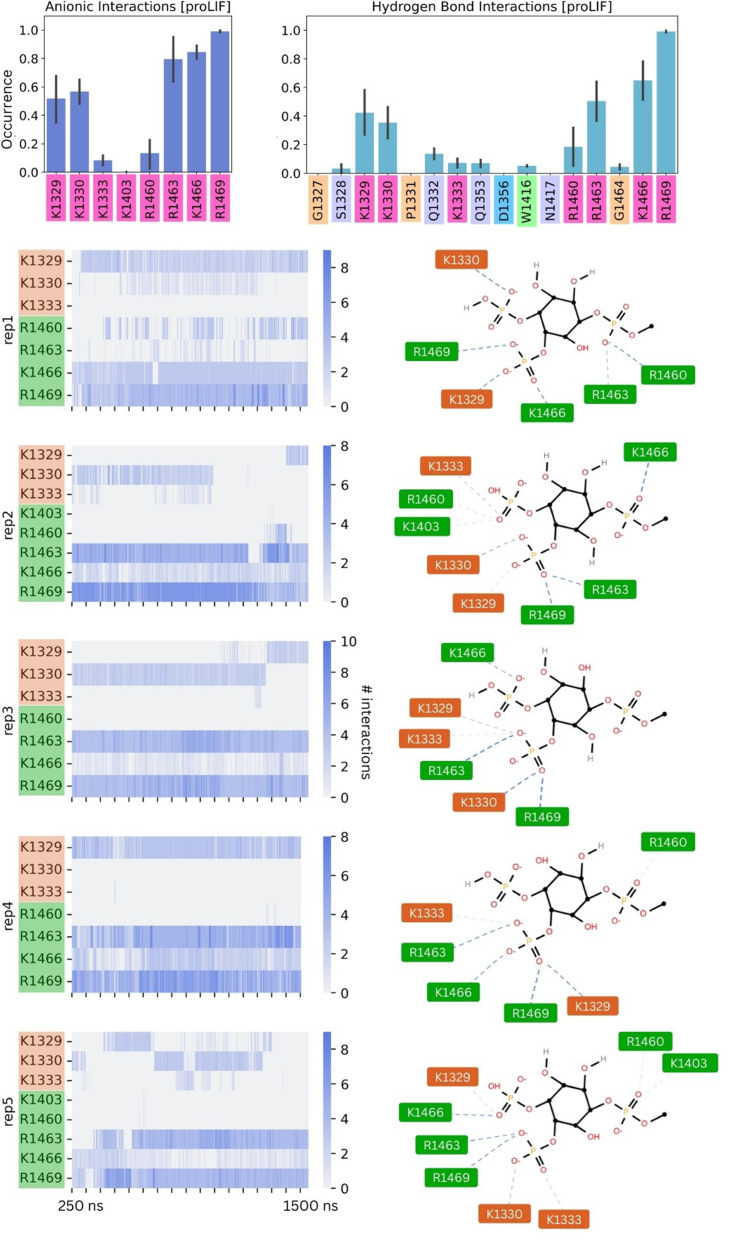
Electrostatic interactions between the PI(4,5)P_2_ headgroup and binding residues. ProLIF bar charts describing the occurrence of anionic and hydrogen bond interactions between the PI(4,5)P_2_ headgroup and binding residues; error bars show standard error, n = 5 (top). Per replicate data, showing the number of electrostatic interactions detected between the PI(4,5)P_2_ headgroup and each of the binding residues (left); atomic interactions summarized in a ProLIF LigPlot (right).

**Figure 4—figure supplement 3.**
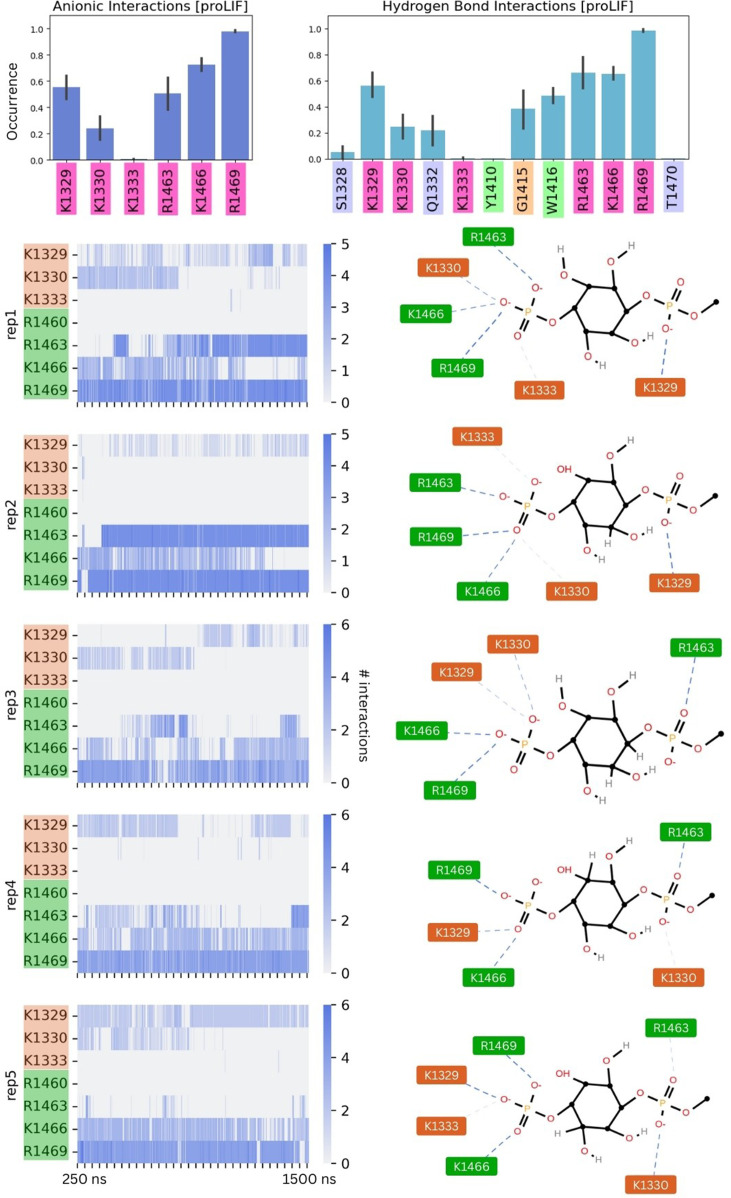
Electrostatic interactions between the PI(4)P_1_ headgroup and binding residues. ProLIF bar charts describing the occurrence of anionic and hydrogen bond interactions between the PI(4)P_1_ headgroup and binding residues; error bars show standard error, n = 5 (top). Per replicate data, showing the number of electrostatic interactions detected between the PI(4)P_1_ headgroup and each of the binding residues (left); atomic interactions summarized in a ProLIF LigPlot (right).

The 4’-phosphate formed interactions with residues belonging to the DIII–IV linker (K1329 and K1333) and DIV S4–S5 linker (K1666 and R1469) with similar frequency, in 55–60% of simulation frames (Figure 4B). In contrast, the 5’-phosphate formed contacts with three VSD-IV S4–S5 residues (R1463, K1466, and R1469) in 70–98% of simulation frames and DIII–IV linker residues in 37–48% of frames. Taken together, this data suggests that although the headgroup is flexible when bound, the 5’-phosphate is more important for coordinating VSD-IV S4–S5 residues while the 4’-phosphate associates with both regions of the binding site.

Atomistic simulations of PI(4)P bound from the VSD-III side show that this is also a stable pose (RMSD <2 Å), where the headgroup interacts with the same positively charged residues as seen for PI(4,5)P_2_ (Figure 4A, Figure 4—figure supplement 1). The residues on the S4–S5 linker, R1463, K1466, and R1469, predominantly bind to the 4’-phosphate (Figure 4B). Due to the more buried location of PI(4)P binding, the PO4 phosphate can associate more with DIII–IV linker lysines (Figure 4B), however, the absence of the 5’-phosphate leads to a reduced number of total electrostatic interactions (Figure 4—figure supplement 3).

To investigate structural changes that might occur in the presence of PI(4)P/PI(4,5)P_2_, we also carried out simulations of the inactivated Naᵥ1.4 structure without any PIPs bound for comparison. Although the DIII–IV linker remains bound throughout simulations both with and without PIPs, residues P1336 and R1337 in the DIII–IV linker downstream of the IFM motif are significantly less mobile with PI(4,5)P_2_ present (Figure 4C). Additionally, seven residues belonging to the DIV S4–S5 linker, including binding residues K1466 and R1469, also have significantly lower mobility when PI(4,5)P_2_ is bound (Figure 4C). In the PI(4)P bound simulations, there were no significant differences in DIII–IV linker or S4–S5 linker mobility.

To further probe whether PIP can stabilize the DIII–IV linker and the inactivation gate, we applied coarse-grained simulations with the DIII–IV linker unrestrained. In simulations of WT Naᵥ1.4, the IFM has a reduced stability within its binding pocket when PIP is excluded from the membrane (Figure 4E). To accentuate this effect, we simulated an inactivation-deficient variant of Naᵥ1.4, where the IFM motif is mutated to IQM (Liu et al., 2023). We find that the IQM motif has a greater probability of being tightly associated with the receptor pocket in the presence of PIP compared to without it (Figure 4E), supporting our observation that PIP can stabilize the channel in the inactivated state. This suggests that the presence of PIP may partially rescue some of the structural defects associated with inactivation dysfunction in Naᵥ mutants.

**Figure 5.**
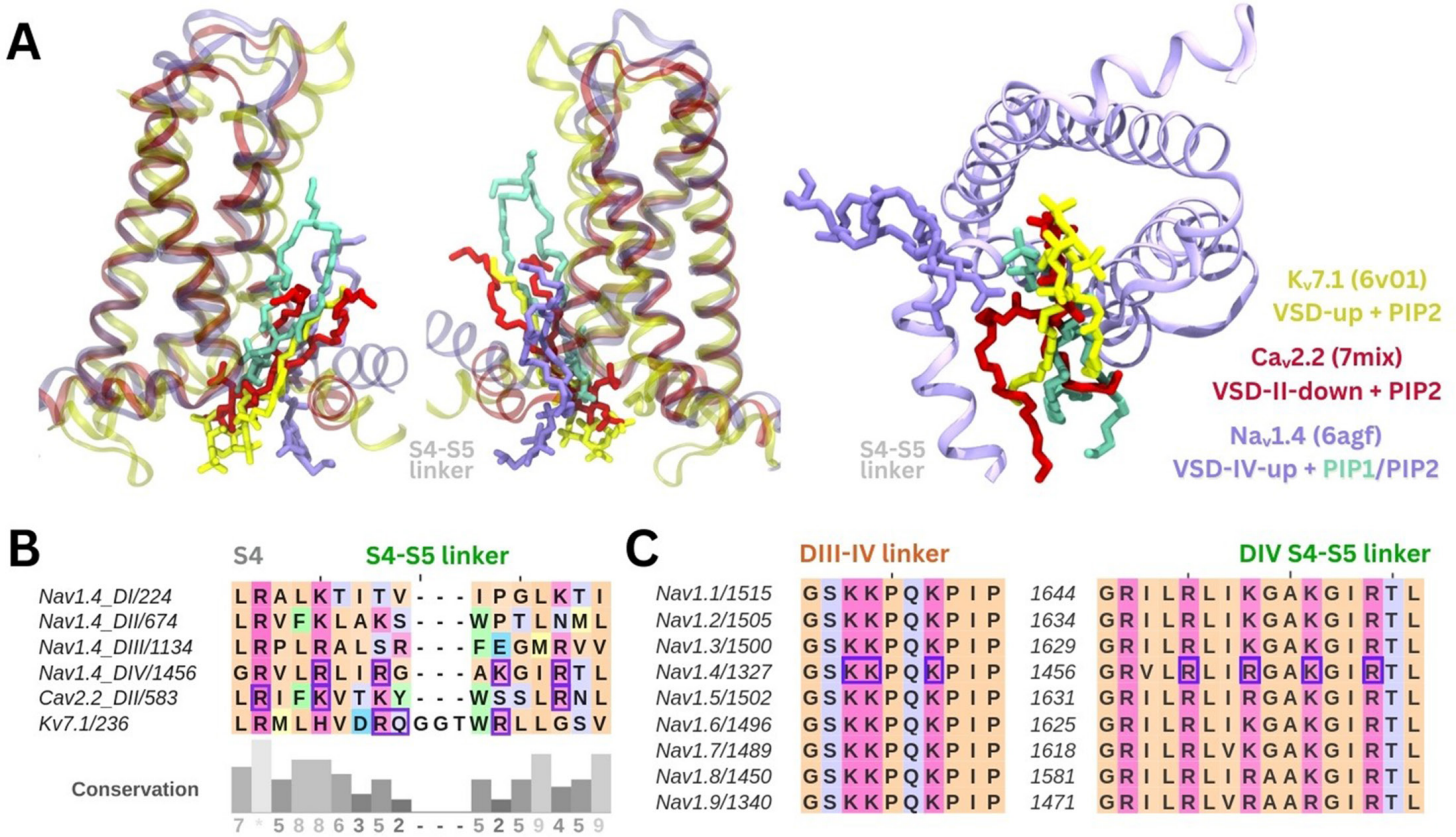
Comparison of the identified phosphoinositide binding site to Naᵥ subtypes and other ion channels. (**A**) Binding poses of PI(4,5)P_2_ (in purple) and PI(4)P (in cyan) aligned with two other tetrameric channels structures Kᵥ7.1 (6v01, in yellow) and Caᵥ2.2 (7mix, in red) that were resolved with PI(4,5)P_2_ at their respective voltage-sensing domains (VSDs). (**B**) Sequence alignment of the S4 helix and S4–S5 linker of the four domains of Naᵥ1.4, compared to VSD-II of Caᵥ2.2 and one of the four identical VSDs of Kᵥ7.1; residues colored by amino acid class; purple boxes indicate PI(4,5)P_2_ binding residues (identified with 5 Å of the headgroup). (**C**) Sequence alignment of the nine human Naᵥ channel subtypes shows high sequence similarity in the S4 helix, S4–S5 linker, and DIII–IV linker regions.

The PIP binding poses seen in our simulations are similar to resolved binding poses for PI(4,5)P_2_ in cryo-EM structures of Caᵥ2.2 and Kᵥ7.1 (Figure 5A). A sequence alignment of Naᵥ1.4 VSDs shows that there are more positively charged residues present in the S4/S4–S5 linker regions of DIV compared to the other domains (Figure 5B). PIP is bound at similar VSD residues on both these ion channels, with PIP forming interactions with a gating charge further up the S4 helix in Caᵥ2.2 due to the VSD being in a different state. Additionally, the high sequence similarity in the S4/S5–S5 linker and DIII–IV linker regions between the nine human Naᵥ channel subtypes suggests conservation of the PIP binding site (Figure 5C).

**Figure 6.**
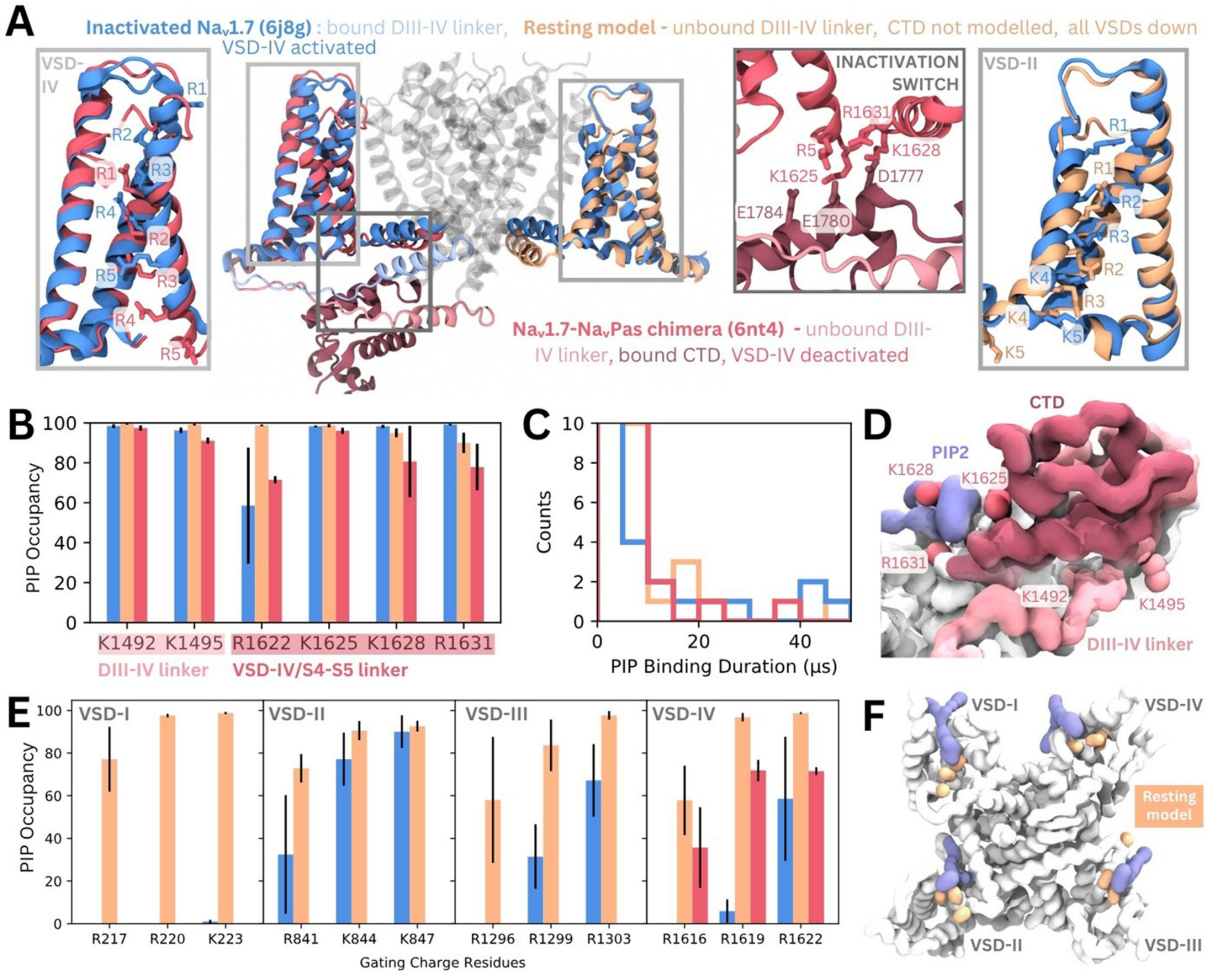
Phosphoinositide (PIP) binding to Naᵥ1.7 with different voltage-sensing domain (VSD) states in coarse-grained simulations. (**A**) Atomistic representation of the three different Naᵥ1.7 structures simulated: (1) the inactivated state (blue, PDB ID: 6j8g) with the VSDs all in the activated, up state, (2) the Naᵥ1.7-NaᵥPas chimera (pink, PDB ID: 6nt4) with the Naᵥ1.7 VSD-IV in the deactivated, down state and a bound NaᵥPas C-terminal domain (CTD), (3) a Naᵥ1.7 resting state model (orange, model generation detailed in Materials and methods) with all four VSDs in the deactivated, down state. Panel insets show the different conformations of VSD-IV (left) and VSD-II (right) across different structures. The inactivation switch formed by the CTD and VSD-IV S4–S5 linker proposed by Clairfeuille et al. is shown (middle). (**B**) Combined occupancy of all PIP species (PIP1, PIP2, PIP3) at binding site residues in the three systems; error bars show standard error, n = 3. (**C**) Distribution of PIP binding durations at the identified site. (**D**) Intracellular view of CTD covering the resting state VSD-IV. Representative snapshot of PIP binding at DIV S4–S5 linker in the Naᵥ1.7-NaᵥPas system. The CTD (dark pink) prevents PIP access to DIII–IV linker lysines, K1492 and K1495. (**E**) Combined PIP occupancy at the bottom three gating charges on VSD I–III in the inactivated (blue) and resting state model (orange) simulations. For VSD-IV, PIP occupancy in the Naᵥ1.7-NaᵥPas system (pink) is also shown. (**F**) Representative simulation snapshot showing PIP (purple) binding at the gating charges (orange) in the resting state model simulations.

To assess possible state- and subtype-dependent differences in PIP binding, we simulated three structures of Naᵥ1.7 with different VSD conformations in a PIP-enriched membrane (Figure 6A). The inactivated Naᵥ1.7 structure (blue, PDB ID: 6j8g) contains all VSDs in the activated/up state and a bound DIII–IV linker. We also simulated a NaᵥPas (American cockroach) chimera structure with a human Naᵥ1.7 VSD-IV in the deactivated/down state (VSDs I–III are activated, and from NaᵥPas) (pink, PDB ID: 6nt4). This structure (Clairfeuille et al., 2019) features a dissociated DIII–IV linker and a resolved CTD bound to the DIV S4–S5 linker (forming the ‘inactivation switch’) at residues identified to form part of our PIP binding site. To assess PIP binding at VSD I–III in the deactivated/down state, we modeled the Naᵥ1.7 resting state based on templates structures in which different VSDs have been captured in deactivated states with the aid of toxins. These structures feature three or more of the gating charge residues below the hydrophobic constriction site (HCS) and displacement of the S4–S5 linker (Figure 6—figure supplement 1). Given that no resting state mammalian Naᵥ channel structure has been resolved, it is possible that the modeled VSDs may not reflect the fully deactivated state.

**Figure 6—figure supplement 1.**
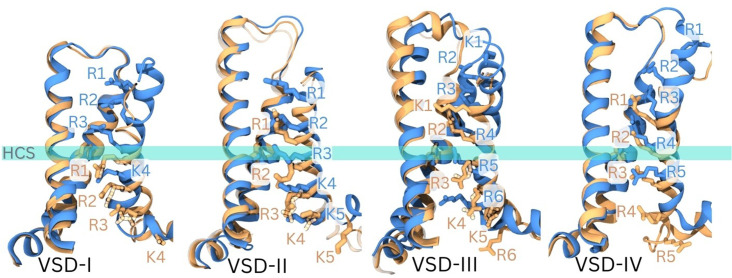
Naᵥ1.7 resting state model validation. Each of the four voltage-sensing domains (VSDs) aligned separately, comparing the inactivated structure (6j8g, in blue) to the resting state model. All the S4 gating charges and the PHE/TYR residue on S2 to indicate the hydrophobic constriction site (HCS) for each VSD (in stick representation). The templates that each corresponding VSD was modeled from are shown in translucent representation. S3 helix hidden for better visualization of gating charge positions.

**Figure 6—figure supplement 2.**
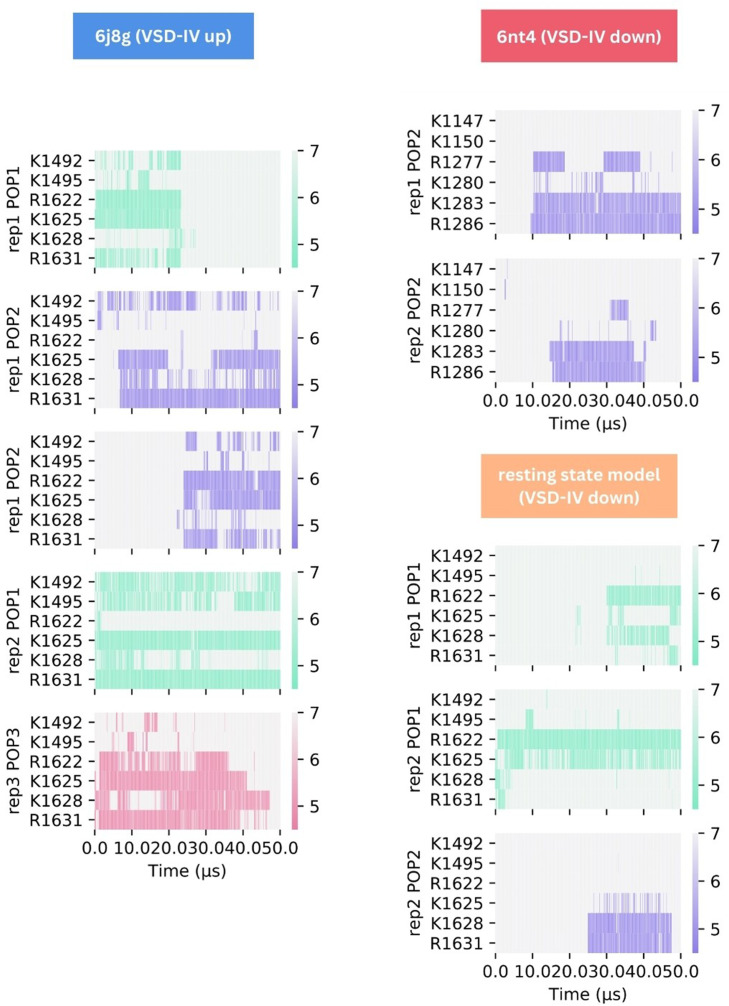
Minimum distance between binding residues on Naᵥ1.7 and bound phosphoinositides (PIPs) shown for long duration binding events (>20 µs) in each system, colored by distance and the type of PIP bound: PIP1 (blue-green), PIP2 (purple), and PIP3 (pink). Five long-term binding events for the inactivated structure of Naᵥ1.7 (PDB ID: 6j8g) are shown on the left. Naᵥ1.7-NaᵥPas chimera (top right, PDB ID: 6nt4) and Naᵥ1.7 resting state model (bottom right) have fewer longer term interactions with PIP at this site. These interactions do not involve residues belonging to the DIII–IV linker (K1147/1492 and K1150/1495).

**Figure 6—figure supplement 3.**
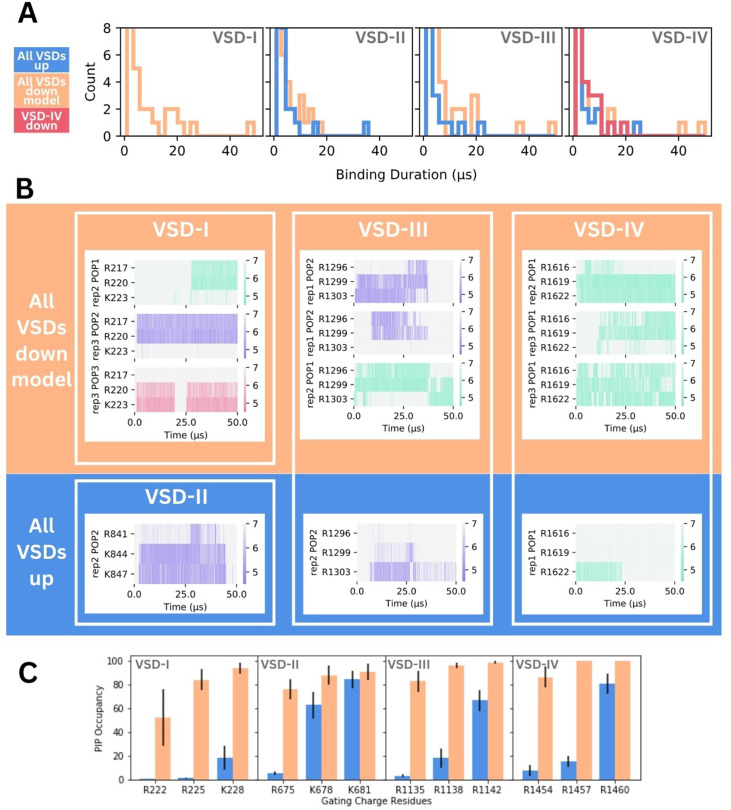
Phosphoinositide (PIP) binding at S4 gating charges. (**A**) Binding duration distributions for PIP binding events at residues belonging to the bottom three gating charges in the S4 helix of voltage-sensing domain (VSD) I–III are shown for the inactivated structure of Naᵥ1.7 (PDB ID: 6j8g, all VSDs up) and resting Naᵥ1.7 model (all VSDs down, C-terminal domain [CTD] not modeled). For VSD-IV, the distribution for the Naᵥ1.7-NaᵥPas chimera (VSD-IV down, CTD bound) is also shown. (**B**) Minimum distance between terminal three gating charges on each VSD and bound PIP for long duration (>20 µs) binding events shown for the resting Naᵥ1.7 model (top) and inactivated Naᵥ1.7 structure (bottom). (**C**) Combined PIP occupancy at the bottom three gating charges on VSD I–IV in the inactivated (blue) and resting state model (orange) simulations of a Naᵥ1.4 resting state model; error bars show standard error, n = 3.

In triplicate 50 µs coarse-grained simulations, PIPs bind to the analogous site to that seen in inactivated Naᵥ1.4 in the inactivated Naᵥ1.7 structure, interacting with residues belonging to both the DIII–IV linker and VSD-IV for durations comparable to Naᵥ1.4, (Figure 6B–C, Figure 6—figure supplement 2). Binding of PIP to the DIV S4–S5 linker to the deactivated VSD-IV in the Naᵥ1.7-NaᵥPas chimera and resting state model was also observed (Figure 6B–C). However, in the Naᵥ1.7-NaᵥPas chimera, the PIP bound at the S4–S5 linker cannot simultaneously associate with the DIII–IV linker (Figure 6—figure supplement 2), due to its sequestration by the CTD, which moves the lysines away from the binding VSD-IV residues (Figure 6D). Instead, K1491, K1492, and K1495 (on the DIII–IV linker) are occupied by different PIPs on the other side of the VSD. In the resting state model, which features an unbound DIII–IV linker PIPs binding at the DIV S4–S5 residues also do not associate with the DIII–IV linker (Figure 6—figure supplement 2). Comparison of binding durations at this site across the three systems reveals a greater number of long PIP interactions (>20 µs) with inactivated Naᵥ1.7 (Dib-Hajj et al., 2013) compared to Naᵥ1.7-NaᵥPas (Venance et al., 2006) or the resting state model (Mantegazza et al., 2021) where VSD-IV is down, and the DIII–IV linker is dissociated.

There are state-dependent differences in PIP occupancy at the gating charges in each VSD (Figure 6E–F, Figure 6—figure supplement 3A and B). PIP can associate with the lowest three gating charges VSD-I of the resting state model, but not in the inactivated state (Figure 6E). This is likely due to the large displacement in the DI-S4 helix, which moves down three helical turns in the resting state model so that all gating charges are below the HCS (Figure 6—figure supplement 1). In VSD-II and -III, the PIP interaction differences between inactivated and resting state are present but less pronounced, owing to a smaller difference in the relative displacement between the gating charges between states (Figure 6—figure supplement 1). PIP occupancy is also higher at VSD-IV when it is in the deactivated conformation, however, the presence of a bound CTD as seen in the Naᵥ1.7-NaᵥPas model reduces this occupancy of PIP (Figure 6E). Additional simulations of a resting state model of Naᵥ1.4 built using our Naᵥ1.7 resting state model as a template suggest that similar gating charge interactions occur for Naᵥ1.4 when the VSDs are deactivated (Figure 6—figure supplement 3C).

## Discussion

Recently, PI(4,5)P_2_ was shown to be a negative regulator of Naᵥ1.4, modulating channel kinetics and voltage dependence. Presence of PI(4,5)P_2_ causes a depolarizing shift in the voltage dependence of activation, that is a stronger stimulus is required to produce Naᵥ1.4 opening. Additionally, it stabilizes the inactivated state of Naᵥ1.4, marked by both shortened times to inactivation and slowed recovery from the inactivation.

Using a multiscale simulation approach, we identified a putative PIP binding site comprised of positively charged residues belonging to the S4 helix/S4–S5 linker of VSD-IV (R1460, R1463, K1466, R1469) and DIII–IV linker (K1329, K1330, K1333, K1352). Coarse-grained simulations of Naᵥ1.4 embedded in a complex membrane showed that PIP interacts with residues belonging to VSD-IV and the DIII–IV linker. In coarse-grained enriched PIP simulations, PIP2 formed longer duration interactions with Naᵥ1.4 than PIP1 and PIP3, supported by a greater number of charged interactions. Atomistic simulations verified the stability of PI(4,5)P_2_ (the most common PIP2 species in the plasma membrane) at this site and showed that the binding of PI(4,5)P_2_ reduces the mobility of some DIV S4–S5 and DIII–IV linker residues. Simulations of Naᵥ1.7 with VSDs in different conformational states showed that the PIP binding site is conserved in Naᵥ1.7 and that PIP interactions at VSD gating charges are functional state dependent, with more interactions being formed when the VSDs are deactivated.

The DIII–IV linker, CTD, and S4–S5 linkers all play key roles throughout the Naᵥ conformational cycle. Mutation of the IFM inactivation motif as well as other residues in the DIII–IV linker alter fast inactivation and recovery from fast inactivation (McPhee et al., 1998; West et al., 1992). While the precise role of the Naᵥ CTD and its conformation during the Naᵥ activation cycle remain elusive, it is likely to be important for coordinating fast inactivation (Clairfeuille et al., 2019; Mantegazza et al., 2001). CTD binding to DIV S4–S5 and sequestration of the DIII–IV linker is proposed to occur in the resting state. After the pore opens, activation of VSD-IV is thought to cause CTD dissociation, releasing the DIII–IV linker to allow fast inactivation. Residues in ‘switch 1’ of the CTD binding site on the DIV S4–S5 stably bind PIP in our simulations of inactivated Naᵥ1.4 and Naᵥ1.7. We hypothesize that PIP binding at this location makes it more difficult for the CTD to reassociate with VSD-IV, a conformational change which is required during recovery from inactivation.

More generally, the S4–S5 linkers in all four domains couple the VSD to the pore helices and adopt different orientations depending on VSD activation states. When the VSD is activated (in the open and inactivated states), the S4–S5 linkers lie parallel to the membrane. In the resting state, when the voltage sensor is deactivated, the S4–S5 linkers move downward below the plane of the membrane. We propose that PIP binding at the identified site could additionally stabilize both the DIV S4–S5 linker and DIII–IV linker to favor the inactivated state. Although the recovery from fast inactivation occurs on the order of several milliseconds (Bezanilla and Armstrong, 1977), and is beyond atomistic simulation timescales, we observed statistically significant reductions in the RMSF of several DIII–IV linker and DIV S4–S5 linker residues when PI(4,5)P_2_ was bound in 1.5 µs atomistic simulations. Reduction in the mobility of the DIII–IV linker may slow the dissociation of the upstream IFM motif and stabilization of the S4–S5 linker prevents the downward movement required for the channel to transition back to the resting state.

The PIP binding residues identified here are conserved in Naᵥ1.1–1.9 (Figure 5C), suggestive of a shared binding site and mechanism for PIP-mediated modulation across subtypes. Mutations at these conserved residues in other subtypes lead to various gain-of-function diseases (Table 1). For example, analogous to the Naᵥ1.4 R1469 residue, the R1642C mutation (in Naᵥ1.3) leads to developmental epileptic encephalopathy (Zaman et al., 2018), and R1644C/H mutations in Naᵥ1.5 (analogous to R1469 in Naᵥ1.4) cause cardiac arrythmias, characterized by accelerated rates of channel recovery from inactivation (Frustaci et al., 2005). Consistent with our observations, these diseases with mutations on the DIII–IV linker are likely to reduce PI(4,5)P_2_ binding, which could be a contributing factor to instability of inactivated state in these pathogenic variants. These inactivation-deficient variants, as well as the IQM variant that we simulated, further emphasize that interactions between PI(4,5)P_2_ with Naᵥ channels could be important for prolonging the fast-inactivated state.

**Table 1.** Disease causing point mutations at analogous phosphoinositide (PIP) binding residues in Naᵥ subtypes (described in the UniProt database).

Naᵥ1.4 residue #	Analogous residue #	Subtype	Disease information; mechanism
K1330	K1505N	Naᵥ1.5	Long QT3 syndrome; unknown significance
R1463	K1641N	Naᵥ1.2	Benign familial infantile seizure; unknown significance
R1469	R1657C	Naᵥ1.1	Generalized epilepsy with febrile seizures plus; depolarizing shift in voltage dependence of activation, reduced current, accelerated recovery from slow inactivation
	R1642C	Naᵥ1.3	Developmental epileptic encephalopathy; accelerated recovery from inactivation
	R1644C R1644H	Naᵥ1.5	Long QT3 syndrome Brugada syndrome

The PIP binding site identified here harbors sequence and structural similarity to PI(4,5)P_2_ binding sites found in other cation channels (Figure 5B). For example, PI(4,5)P_2_ is resolved at a similar site near the VSD and S4–S5 linker in a recent cryo-EM structure of Kᵥ7.1, where the phosphate headgroup forms analogous contacts to R249 and R243 (PDB ID: 6v01) (Sun and MacKinnon, 2020). Despite differences in the role of PI(4,5)P_2_, which negatively regulates Naᵥ1.4 but is required for Kᵥ7.1 pore opening, the binding site appears to be conserved. Based on the PI(4,5)P_2_ binding site, a structurally similar compound was developed as an activator of Kᵥ7 channels and proposed to be a future antiarrhythmic therapy (Liu et al., 2020).

PI(4,5)P_2_ also binds to the down, deactivated state of VSD-II in Caᵥ2.2 (PDB ID: 7mix) (Gao et al., 2021). In this structure, the PI(4,5)P_2_ headgroup interacts with two VSD-II gating charges, R584 and K587. Compared to the positioning of PI(4,5)P_2_ in our simulations of Naᵥ1.4 with an activated VSD-IV, the headgroup associates further up the S4 helix in Caᵥ2.2 due to the VSD being in a deactivated state. This is also seen in Kᵥ7.1 which contains an extended GGT loop in the S4–S5 linker which prevents PI(4,5)P_2_ binding in the VSD-down state (Mandala and MacKinnon, 2023). In our coarse-grained simulations of the resting Naᵥ1.7 model, we observe a similar state-dependent difference in PI(4,5)P_2_ interactions with the deactivated states of each VSD. Since activation of VSDs I–III are known to be coupled to channel opening (Goldschen-Ohm et al., 2013), we propose that PIP binding at these VSDs impedes their ability to activate and thus increases the voltage threshold required of opening. PI(4,5)P_2_ binding at VSD-IV is prevented by the presence of the CTD (and absence of the DIII–IV linker) in the resting state, thus not affecting the kinetics of inactivation onset.

The leftward shift in voltage dependence of inactivation is less pronounced when PI(4,5)P_2_ is converted to PI(4)P rather than completely dephosphorylated to PI (Gada et al., 2023). This suggests that PI(4)P may play a compensatory role when PI(4,5)P_2_ is not present. This is supported by our simulations which show that PI(4)P can also stably occupy the identified binding site, albeit with shorter duration and form less electrostatic interactions compared to PI(4,5)P_2_. Our atomistic simulations also showed that the 5’-phosphate is more important than the 4’-phosphate for forming interactions with the DIV S4–S5 linker residues of the inactivation switch. These factors suggest that PI(4,5)P_2_ binding is preferred over PI(4)P at this site and can better compete to bind over the CTD, implying that PI(4,5)P_2_ is more effective at stabilizing the inactivated state and inhibiting recovery to the resting state.

Simulations using the Martini2.2 forcefield have previously been used to investigate lipid-protein interactions (Corradi et al., 2018) and successfully predict specific lipid binding sites, including the PIP binding site on K_ir_ channels (Stansfeld et al., 2009). While the Martini2.2 PIP species can be parameterized for a specific sub-species (e.g. PI(3,4)P_2_ vs PI(4,5)P_2_), we instead employed atomistic simulations to complement and strengthen findings from coarse-grained simulations, allowing us to identify the specific contribution of the 4’- and 5’-phosphate groups to binding as well as to investigate the conformational changes associated with PI(4,5)P_2_ binding. Since the precise protonation of the PI(4,5)P_2_ headgroup in a physiological setting is unclear, we explore one case with the 4’-phosphate protonated. Given that the PI(4,5)P_2_ headgroup can adopt slightly different binding orientations and can fluctuate over the course of atomistic simulations (Figure 4—figure supplement 2), we expect the alternate protonation state to have similar affinity for the binding site.

In this work we made use of the Martini2.2 model for our coarse-grained simulations, however recently the refined Martini3 (Souza et al., 2021) has become available and will be a useful tool for further interrogating protein-lipid interactions, as a greater number of lipid parameters become available. Our coarse-grained simulations also allow us to investigate the association of other lipid types with Naᵥ1.4. While we focus on PIP here, there are other lipid species that have modulatory effects on Naᵥ channels, such as cholesterol (Amsalem et al., 2018), glycolipids, DG, LPC, and PI, and their interactions warrant further investigation.

### Conclusion

**Figure 7.**
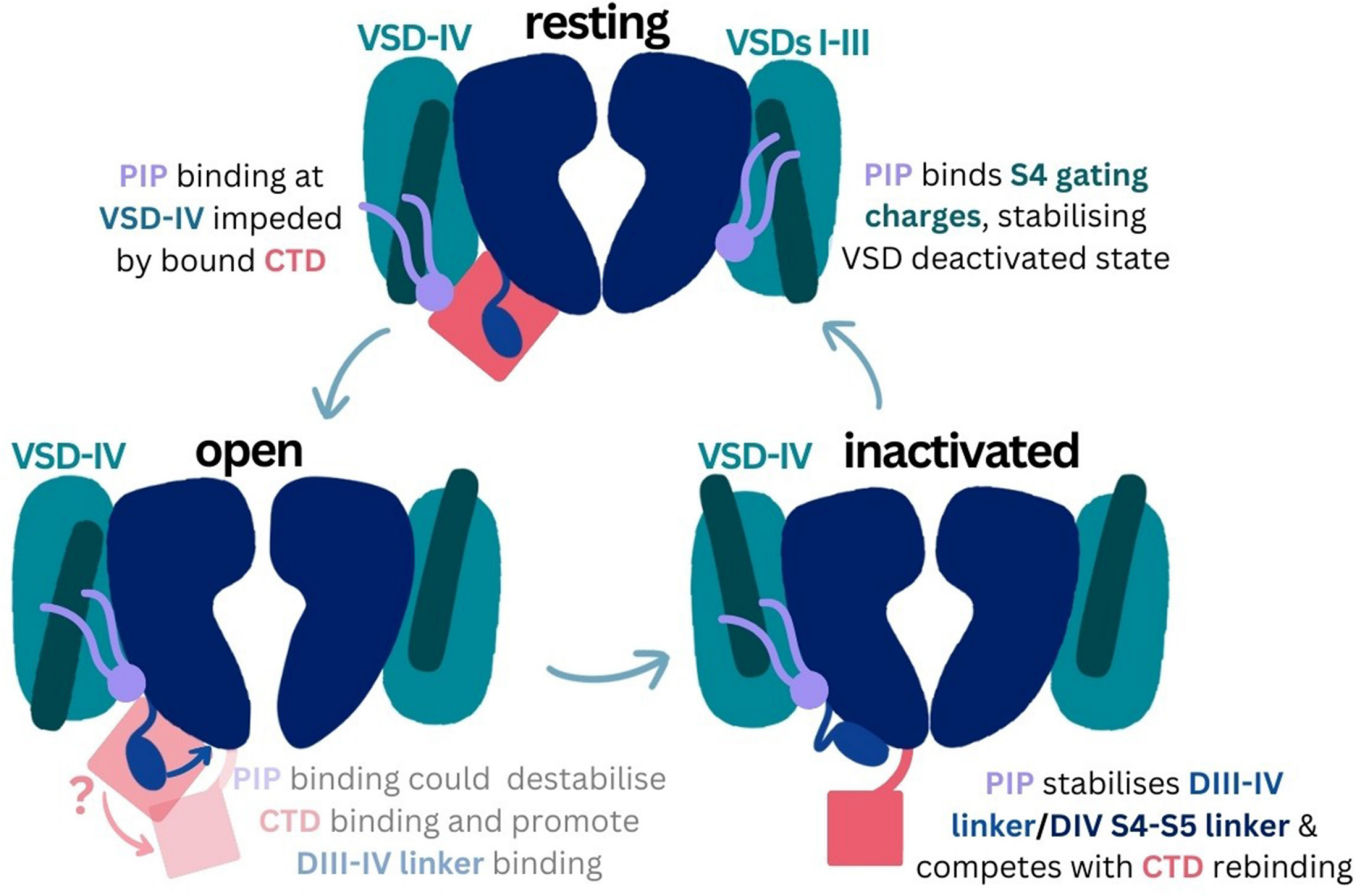
Proposed mechanism of phosphoinositide (PIP) effects on the sodium channel functional cycle.

Using multiscale simulations, we show that PI(4,5)P_2_ binds stably to inactivated structures of Naᵥ1.4 and Naᵥ1.7 at a conserved site within the DIV S4–S5 linker. As the CTD is proposed to also bind here during recovery from inactivation, we hypothesize that PI(4,5)P_2_ competes with the CTD to bind to this site, prolonging inactivation. At this site, PI(4,5)P_2_ simultaneously binds to the DIII–IV linker which is responsible for allosterically blocking the pore during fast inactivation (Figure 7). Its binding reduces the mobility of both the DIV S4–S5 and DIII–IV linkers, potentially slowing the conformational changes required for the channel to recover to the resting state. We also propose that in the resting state, PIPs form additional interactions with S4 gating charges, particularly in VSD-I, anchoring them to the membrane in a way which may make the upward movement required for their activation more difficult. Our results provide insight into how sodium channels are modulated by PIPs, an important step for the development of novel therapies to treat Naᵥ-related diseases.

## Materials and methods

### Coarse-grained simulations

Coarse-grained simulations of Naᵥ1.4 embedded in a complex mammalian membrane were carried out to investigate lipid-protein interactions. The inactivated Naᵥ1.4 alpha subunit (PDB ID: 6agf) (Pan et al., 2018) was coarse-grained using the CHARMM-GUI Martini Maker (Jo et al., 2008; Qi et al., 2015) and embedded in a 360 Å × 360 Å complex membrane using insane.py (Wassenaar et al., 2015). The composition of the complex mammalian membrane is as reported in Ingólfsson et al., 2014. Three replicate simulations, each with different starting coordinates, were carried out for 16 µs each.

To better sample binding events, we also carried out PIP-enriched simulations in which Naᵥ1.4 was embedded in a 160 Å × 160 Å POPC membrane with 5% of each PIP species, PIP1, PIP2, PIP3 (with charge parameters of –3e, –5e, and –7e, respectively), added to the cytoplasmic leaflet using insane.py. Ten replicate simulations, each with different starting coordinates, were carried out for 80 µs each. To validate our proposed binding site, we additionally mutated the positively charged PIP binding site residues K1329, K1330, K1330, K1352, R1460, R1463, K1466, and R1469 to leucines (‘8L’) or glutamates (‘8E) and simulated these mutant channels in enriched PIP membranes for 20 µs in triplicate. To explore the possibility for PIP to stabilize the DIII–IV linker in an inactivation-deficient Naᵥ1.4 variant, additional coarse-grained simulations were carried out where the DIII–IV linker region (residues L1305-K1341) was unrestrained for both the WT Naᵥ1.4 and simulations in which the IFM motif was mutated to IQM. In these simulations, an elastic network was applied to E1314-G1327 in the linker to preserve the helicity of this region. Flexible linker simulations were conducted in triplicate for 20 µs in both PIP-enriched bilayers and POPC-only bilayers.

To explore possible state- and subtype-dependent differences in PIP binding, the inactivated Naᵥ1.7 structure (PDB ID: 6j8g) (Shen et al., 2019) and the Naᵥ1.7-NaᵥPas chimera with CTD bound and VSD-IV in the deactivated state (PDB ID: 6nt4) (Clairfeuille et al., 2019) were also coarse-grained and simulated in PIP-enriched membranes (same protocol as above) for three replicates of 50 µs each. Additionally, a model Naᵥ1.7 with all four VSDs in the deactivated state was built using Modeller (Sali and Blundell, 1993) and simulated in triplicate for 50 µs each. The template and structural information for this model are detailed in Table 2. In brief, VSD-I down was modeled from 7xve (Huang et al., 2022), VSD-II and VSD-III were both modeled from the deactivated VSD-II from 7k48 (Wisedchaisri et al., 2021), and VSD-IV and the unbound DIII–IV linker were modeled from the corresponding regions in 6nt4 (Clairfeuille et al., 2019). Adjacent S5/S6 regions to each VSD were also modeled from each specified template to ensure proper contacts between the pore domain and VSDs. The CTD was not included in the model. Using these Naᵥ1.7 templates, the resting state model of Naᵥ1.4 was generated and simulated for three replicates in coarse grain with the protein backbone restrained, in a PIP-enriched membrane for 50 µs.

**Table 2.** Generation of the resting state model using a combination of multiple templates for different voltage-sensing domains (VSDs) and pore domains (PDs) of Naᵥ1.7.

Template pdb	Domains used	Res IDs (from template)	Res IDs (human Naᵥ1.7 numbering)	Template info
7xve	VSDI, PDI, PDII	1–404, 541–650	8–411, 864–972	Naᵥ1.7 mutant (L866F, T870M, and A874F on S5II; V947F, M952F, and V953F on S6II; and V1438I, V1439F, and G1454C on S6III, E156K on S2I and G779R on S2II) VSDI deactivated (VSDII partially deactivated but not used)
7k48	VSDII, PDII, VSDIII, PDIII, PDIV	405, 938, 1115–1244	728–973, 1175–1462, 1639–1768	Naᵥ1.7/NaᵥAb chimera, where top half of each VSD is Naᵥ1.7 VSDII; all VSDs deactivated with engineered tarantula toxin m3-Huwentoxin-IV bound
6nt4	VSDIV, PDIV, PDI	939–1244, 239–404	1463–1768, 246–411	Naᵥ1.7/NaᵥPas chimera, full Naᵥ1.7 VSDI, and VSDIV deactivated with α-scorpion neurotoxin AaH2 bound
7w9k	Loops between S5 and S6 regions for PDII, PDIII, and PDIV	563–619, 811–918, 1141–1213	886–942, 1335–1442, 1665–1737	Fast-inactivated Naᵥ1.7 (all VSDs activated) DIII–IV linker bound to pore

All systems were solvated and ionized with 150 mM NaCl. All coarse-grained simulations were carried out with GROMACS 2022 (Bauer et al., 2023) using the Martini2.2 forcefield (de Jong et al., 2013) and the PIP parameters for each charge state, where PIP1 is based on PI(3)P and PIP2 is based on PI(3,4)P2 (López et al., 2013). Energy minimization was carried out on each system using the steepest descent method for 1000 steps. Following this, equilibration in the constant pressure, constant volume (NVT) ensemble at 1 atm for 10 ps was carried out with backbone position restraints (1000 kJ mol^−1^nm^−2^) using a 2-fs time step. Following this, constant pressure and temperature (NPT) equilibration simulations were carried out, using 5, 10, and 20 fs time steps in sequence, with each running for 5000 steps. 1 atm pressure was maintained using a Berendsen barostat with semi-isotropic conditions. Production simulations were carried out in the NPT ensemble, kept at a temperature of 310 K using the Nose-Hoover thermostat (Evans and Holian, 1985) and a pressure of 1 bar using the Parrinello-Rahman barostat (Parrinello and Rahman, 1981). A time step of 20 fs was used. During production simulations, the backbone beads were weakly restrained to their starting coordinates using a force constant of 10 kJ mol^−1^ nm^−2^.

### Atomistic simulations

Atomistic simulations were performed to characterize atomic interactions between Naᵥ1.4 residues and the bound PI(4,5)P_2_ headgroup. Frames from a stable PIP2 binding event (from replicate 1 of enriched PIP simulations) were clustered using a selection of the bound PIP2 headgroup beads (C1 C2 C3 PO4 P1 P2) and binding residues K1329, K1330, K1333, K1463, K1466, and R1469 with an RMSD cutoff of 2.5 Å. The protein and bound PIP2 were extracted from the representative frame of the cluster and backbone beads of the coarse-grained VSD-IV were aligned to the corresponding carbon-alpha atoms in the original cryo-EM structure of Naᵥ1.4 (Pan et al., 2018). PIP2 was backmapped to atomistic coordinates of SAPI24 (the CHARMM lipid for PI(4,5)P_2_ with –2e charge on P5, –1e charge on P4, and –1e on the PO4, as shown in Figure 4D) and the protein was replaced with the 6agf structure. The system was embedded in a 140 Å × 140 Å POPC membrane, solvated, and 0.15 M NaCl added using the CHARMM-GUI Membrane Builder (Jo et al., 2008; Wu, 2014; Lee et al., 2016). An identical system was set up with Naᵥ1.4 in a POPC membrane without PIP.

Atomistic simulations were performed with Amber20 (Case, 2020), using the CHARMM36m (Huang et al., 2017) and TIP3P water (Jorgensen et al., 1983) forcefields. Equilibration steps were performed (minimization, heating, pressurizing), with 5 kJ mol^–1^ restraints on the protein backbone, followed by 24 ns of gradually reducing restraints. Five replicates of unrestrained production equilibrium simulations were performed, run for 1.5 µs each. The temperature was set at 310 K using the Langevin thermostat (Loncharich et al., 1992) and at a collision frequency of 5 ps^–1^. Pressure was set at 1 bar using the Monte Carlo barostat (Åqvist et al., 2004) with anisotropic scaling and relaxation time of 1 ps. 12 Å van der Waals cutoff and hydrogen bond SHAKE constraints were used. Hydrogen mass repartitioning was used to enable a 4 fs timestep (Hopkins et al., 2015). PI(4)P_1_ was also simulated with the same atomistic procedures, using the SAPI14 CHARMM lipid (with –2e charge on P4 and –1e on the PO4, as shown in Figure 4D).

### Analysis

Coarse-grained lipid-protein interactions were characterized using in-house python scripts which used the numpy, MDAnalysis (Michaud-Agrawal et al., 2011), and pandas libraries (as done previously in Chang Lin et al., 2022). A cutoff of 0.7 nm was used to define interactions between lipids and protein residues. Distance heatmaps were generated based on the minimum distance between a PIP bead and the side chain (SC2) bead of the interacting arginine or lysine residue. Binding durations were calculated by first counting the number of interactions between each PIP and VSD-IV binding residues (R1460, R1463, K1466, and R1469). A binding event is defined as the time between the first and last time that PIP interacts with two or more binding residues on VSD-IV, if it interacts with a minimum of one binding residue between this period. Stability of IFM/IQM motif binding in flexible linker coarse-grained simulations was assessed by measuring the distance between the center of mass of the phenylalanine/glutamine residue to the center of mass of three residues (L1153, I1485, and N1591) within the IFM receptor site.

For atomistic simulations, MDAnalysis (Michaud-Agrawal et al., 2011) was used to calculate RMSD of various parts of the protein and PIP headgroup, and RMSF of the carbon-alphas. Statistical significance in RMSF was assessed using Student’s t-test. ProLIF (Bouysset and Fiorucci, 2021) was used to compute electrostatic interactions between the protein and binding residues. Representative snapshot of PIP2 binding was generated using the WMC Clustering Tool in Visual Molecular Dynamics (VMD) to identify the top cluster of the PIP2 headgroup (RMSD cutoff of 3 Å), then subsequently cluster the six binding residues to identify the most representative binding configuration. Trajectories were strided every 1 ns and the first 250 ns of simulations was discarded as equilibration time for analyses of RMSF, ProLIF interactions, and clustering. All analysis scripts are available on GitHub (nav_pip_project; copy archived at Tao, 2023).

Simulations were visualized and protein image figures produced using VMD (Humphrey et al., 1996). ClustalOmega (Goujon et al., 2010; Sievers et al., 2011) and JalView (Waterhouse et al., 2009) were used to generate and visualize sequence alignments. Structural representations of Kᵥ7.1, Caᵥ2.2, and Naᵥ1.4 structures were created in VMD by aligning each of the S4 helices on the VSD where the PIP was bound.

## Data Availability

All analysis code is available on GitHub (nav_pip_project; copy archived at Tao, 2023).

## Additional files

MDAR checklist
