# Supplementary material for: A binding site for phosphoinositides described by multiscale simulations explains their modulation of voltage-gated sodium channels
Source: eLife. 2024 Mar 11;12:RP91218. doi: 10.7554/eLife.91218 (PMC10928511; doi:10.7554/eLife.91218)
Supplement: Peer review history

# Editor report: eLife assessment

**DOI:** 10.7554/eLife.91218.3.sa0

**Authors:** Toby W Allen

**Affiliations:** RMIT University Australia

**Keywords:** Convincing, Important

This **important** study employs multiscale simulations to show that PIP2 lipids bind to DIV S4-S5 linkers within the inactivated state of a voltage-gated sodium channel, affecting the coupling of voltage sensors to the ion-conducting pore. The authors demonstrate that PIP2 prolongs inactivation by binding to the same site that binds the C-terminal during recovery from inactivation, and they suggest that binding to gating charges in the resting state may impede activation, both findings that contribute to our understanding of sodium channel modulation. The coarse-grained and atomistic molecular dynamics simulations are **convincing**, including state dependence and linker mutants to back up the claims.

# Referee report: Reviewer #1 (Public Review)

**DOI:** 10.7554/eLife.91218.3.sa1

Summary: Here, the authors were attempting to use molecular simulation or probe the nature of how lipids, especially PIP lipids, bind to a medically-important ion channel. In particular, they look at how this binding impacts the function of the channel.

Strengths: The study is very well written and composed. The techniques are used appropriately, with plenty of sampling and analysis. The findings are compelling, and provide clear insights into the biology of the system.

Weaknesses: A few of the analyses are hard to understand/follow, and rely on "in house" scripts. This is particularly the case for the lipid binding events, which can be difficult to compute accurately. However the provision of these scripts on github means that these can be assessed by the reader if desired. Additionally, a lack of experimental validation, or coupling to existing experimental data, limits the study.

It is my view that the authors have achieved their aims, and their findings are compelling and believable. Their findings should have impacts on how researchers understand the functioning of the Nav1.4 channel, as well as on the study of other ion channels and how they interact with membrane lipids.

# Referee report: Reviewer #2 (Public Review)

**DOI:** 10.7554/eLife.91218.3.sa2

Summary:

Lin Y., Tao E., et al. used multiscale MD simulations to show that PI(4,5)P2 binds stably to an inactivated state of Nav channels at a conserved site within the DIV S4-S5 linker, which couples the voltage sensing domain (VSD) to the pore. The authors hypothesized that PI(4,5)P2 prolongs inactivation by binding to the same site where the C-terminal tail is proposed to bind during recovery from inactivation. They convincingly showed that PI(4,5)P2 reduces the mobility of both the DIV S4-S5 linker and the DIII-IV linker, thus slowing the conformational changes required for the channel to recover to the resting state. They also conducted MD simulations to show that phosphoinositides bind to VSD gating charges in the resting state of Nav channels. These interactions may anchor VDS at the resting state and impede its activation. Their results provide a mechanism by which phosphoinositides alter the voltage dependence of activation and the recovery rate from inactivation, an important step for developing novel therapies to treat Nav-related diseases. However, the study is incomplete lacks the expected confirmatory studies which are relevant to such proposals.

Strengths:

The authors identified a novel binding between phosphoinositides and the VSD of Nav and showed that the strength of this interaction is state-dependent. Based on their work, the affinity of PIPs to the inactivated state is higher than the resting state. This work will help pave the way for designing novel therapeutics that may help relieve pain or treat diseases like arrhythmia, which may result from a leftward shift of the channel's activation.

Weaknesses:

However, the study lacks the expected confirmatory studies relevant to such proposals. For example, one would expect that the authors would mutate the positive residues that they claim to make interactions with phosphoinositides to show that there are much fewer interactions once they make these mutations. Another point is that the authors found that the main interaction site of PIPs with Nav1.4 is the VSD-DIV and DIII-DIV linker. This interaction is expected to delay fast inactivation if it happens at the resting state. The authors should make a resting state model of the Nav1.4 channel to explain the recent experimental data showing that PIP2 delays the activation of Nav1.4, with almost no effect on the voltage dependence of fast inactivation.

The reviewers answered most of my concerns about the first version of the manuscript.

# Referee report: Reviewer #3 (Public Review)

**DOI:** 10.7554/eLife.91218.3.sa3

Summary:

This work uses multiscale molecular dynamics simulations to demonstrate molecular mechanism(s) for phosphatidylinositol regulation of voltage gated sodium channel (Nav1.4) gating. Recent experimental work by Gada et al. JGP 2023 showed altered Nav1.4 gating when Nav1.4 current was recorded with simultaneous application of PI(4,5)P2 dephosphorylate. Here the authors revealed probable molecular mechanism that can explain PI(4,5)P2 modulation of Nav1.4 gating. They found PIP lipids interacting with the gating charges - potentially making it harder to move the voltage sensor domain and altering the channels voltage sensitivity. They also found a stable PIP binding site that reaches the D_IV S4-S5 linker, reducing the mobility of the linker and potentially competing with the C-terminal domain.

Strengths:

Using multiscale simulations with course-grained simulations to capture lipid-protein interactions and the overall protein lipid fingerprint and then all-atom simulations to verify atomistic details for specific lipid-protein interactions is extremely appropriate for the question at hand. Overall, the types of simulation and their length are suitable for the questions the authors pose and a thorough set of analysis was done which illustrates the observed PIP-protein interactions.

Weaknesses:

Although the set of current simulations and analysis supports the conclusions drawn nicely, the course-grained simulations have further utility than that utilized by the authors. With the 4to1 heavy atoms bead mapping in Martini 2 some detailed chemical specificity is averaged out but parameters for different PIP family members do exist - including specific PIP(4,5)P2 vs PIP(3,4)P2, and could have been explored at the course-grained level. However, performing more detailed all-atom simulation, as done in this manuscript, is always advisable to extend and/or confirm course-grained results.

# Author comment: Author Response

**DOI:** 10.7554/eLife.91218.3.sa4

**Authors:** Yiechang Lin, Elaine Tao, James P Champion, Ben Corry

**Affiliations:** Australina National University Canberra Australia; Australian National University Canberra Australia; Australian National University Canberra Australia; Australian National University Canberra Australia

The following is the authors’ response to the original reviews.

We thank the reviewers for their thorough reading and helpful comments which has allowed us to further improve the manuscript. Following the suggestions of the reviewers we have run a number of new simulations including mutations of the PIP binding residues and with an elastic network allowing more mobility of the linker. Together these excellent ideas have allowed us to strengthen the conclusions of the study. Below, we provide point-by-point responses to their suggestions.

**Reviewer #1 (Public Review):**

Summary:

Here, the authors were attempting to use molecular simulation or probe the nature of how lipids, especially PIP lipids, bind to a medically-important ion channel. In particular, they look at how this binding impact the function of the channel.

Strengths:

The study is very well written and composed. The techniques are used appropriately, with plenty of sampling and analysis. The findings are compelling and provide clear insights into the biology of the system.

Weaknesses:

A few of the analyses are hard to understand/follow, and rely on "in house" scripts. This is particularly the case for the lipid binding events, which can be difficult to compute accurately. Additionally, a lack of experimental validation, or coupling to existing experimental data, limits the study.

Our analysis scripts have now been made publicly accessible as a Jupyter notebook on Github https://github.com/etaoster/etaoster.github.io/tree/main/nav_pip_project

It is my view that the authors have achieved their aims, and their findings are compelling and believable. Their findings should have impacts on how researchers understand the functioning of the Nav1.4 channel, as well as on the study of other ion channels and how they interact with membrane lipids.

**Reviewer #2 (Public Review):**

Summary:

Y., Tao E., et al. used multiscale MD simulations to show that PI(4,5)P2 binds stably to an inactivated state of Nav channels at a conserved site within the DIV S4-S5 linker, which couples the voltage sensing domain (VSD) to the pore. The authors hypothesized that PI(4,5)P2 prolongs inactivation by binding to the same site where the C-terminal tail is proposed to bind during recovery from inactivation. They convincingly showed that PI(4,5)P2 reduces the mobility of both the DIV S4-S5 linker and the DIII-IV linker, thus slowing the conformational changes required for the channel to recover to the resting state. They also conducted MD simulations to show that phosphoinositides bind to VSD gating charges in the resting state of Nav channels. These interactions may anchor VDS at the resting state and impede its activation. Their results provide a mechanism by which phosphoinositides alter the voltage dependence of activation and the recovery rate from inactivation, an important step for developing novel therapies to treat Nav-related diseases. However, the study is incomplete and lacks the expected confirmatory studies which are relevant to such proposals.

Strengths:

The authors identified a novel binding between phosphoinositides and the VSD of Nav and showed that the strength of this interaction is state-dependent. Based on their work, the affinity of PIPs to the inactivated state is higher than the resting state. This work will help pave the way for designing novel therapeutics that may help relieve pain or treat diseases like arrhythmia, which may result from a leftward shift of the channel's activation.

Weaknesses:

However, the study lacks the expected confirmatory studies which are relevant to such proposals. For example, one would expect that the authors would mutate the positive residues that they claim to make interactions with phosphoinositides to show that there are much fewer interactions once they make these mutations. Another point is that the authors found that the main interaction site of PIPs with Nav1.4 is the VSD-DIV and DIII-DIV linker, an interaction that is expected to delay fast inactivation if it happens at the resting state. The authors should make a resting state model of the Nav1.4 channel to explain the recent experimental data showing that PIP2 delays the activation of Nav1.4, with almost no effect on the voltage dependence of fast inactivation.

Following the reviewers suggestion we have conducted new simulations demonstrating that there are many fewer protein-PIP interactions after mutating the positive residues as shown in the new Supplementary Fig S6.

The reviewer mentions that if PIPs interact with the VSD-DIV and DIII-DIV linker in the resting state that it could delay fast inactivation. However, as described in the original manuscript and depicted in the schematic (Fig 7) the C-terminal domain impeded PIP binding at the position in the resting state (but not the inactivated state), meaning that PIP does not bind in the resting state to delay fast inactivation. We have clarified this statement in the text on page 14 lines 1-2.

Following the reviewer’s suggestion we have examined PIP binding to a model of the resting state of Nav1.4(in addition to the resting state of Nav1.7 described in the original manuscript) as described on page 12 lines 28-30 (and in Fig S12). Similar to what we saw for Nav1.7, PIP binding to VSDI-III can impair activation of the channel.

Major concern:

(1) Lack of confirmatory experiments, e.g., mutating the positive residues that show a high affinity towards PIPs to a neutral and negative residue and assessing the effect of mutagenesis on binding.

Done as described above

(2) Nav1.4 is the only channel that has been studied in terms of the effect of PIPs on it, therefore the authors should build a resting state model of Nav1.4 and study the effect of PIPs on it.

Done as described above

Minor points:

There are a lot of wrong statements in many areas, e.g., "These diseases 335 are associated with accelerated rates of channel recovery from inactivation, consistent with our observations that an interaction between PI(4,5)P2 and the residue corresponding to R1469 in other Nav 337 subtypes could be important for prolonging the fast-inactivated state." Prolonging the fast inactivated state would actually reduce recovery from inactivation and not accelerate it.

We disagree with this statement from the reviewer which may have come from a misreading of the mentioned sentence. Our statement in the original manuscript is consistent with the original experiments that show that the presence of PIP prolongs the time spent in the fast inactivated state. Mutations at the PIP binding site are likely to reduce PIP binding, and with less PIP bound the channel is expected to recover from inactivation more quickly. We have reworded this sentence for clarity on page 13 line 27-30.

**Reviewer #3 (Public Review):**

Summary:

This work uses multiscale molecular dynamics simulations to demonstrate molecular mechanism(s) for phosphatidylinositol regulation of voltage gated sodium channel (Nav1.4) gating. Recent experimental work by Gada et al. JGP 2023 showed altered Nav1.4 gating when Nav1.4 current was recorded with simultaneous application of PI(4,5)P2 dephosphorylate. Here the authors revealed probable molecular mechanism that can explain PI(4,5)P2 modulation of Nav1.4 gating. They found PIP lipids interacting with the gating charges - potentially making it harder to move the voltage sensor domain and altering the channels voltage sensitivity. They also found a stable PIP binding site that reaches the D_IV S4-S5 linker, reducing the mobility of the linker and potentially competing with the C-terminal domain.

Strengths:

Using multiscale simulations with course-grained simulations to capture lipid-protein interactions and the overall protein lipid fingerprint and then all-atom simulations to verify atomistic details for specific lipidprotein interactions is extremely appropriate for the question at hand. Overall, the types of simulation and their length are suitable for the questions the authors pose and a thorough set of analysis was done which illustrates the observed PIP-protein interactions.

Weaknesses:

Although the set of current simulations and analysis supports the conclusions drawn nicely, there are some limitations imposed by the authors on the course-grained simulations. If those were not imposed, it would have allowed for an even richer set and more thorough exploration of the protein-lipid interactions. The Martini 2 force field indeed cannot change secondary structure but if run with a properly tuned elastic network instead of backbone restraints, the change in protein configuration can be sampled and/or some adaptation of the protein to the specific protein environment can be observed. Additionally, with the 4to1 heavy atoms to a bead mapping some detailed chemical specificity is averaged out but parameters for different PIP family members do exist - including specific PIP(4,5)P2 vs PIP(3,4)P2, and could have been explored.

We thank the reviewer for their excellent suggestions and have run new simulations with an elastic network instead of backbone restraints which have generated new insights. Indeed, as shown in the new panel Fig 4E, the new data allows us to demonstrate that the presence of PIP in the proposed binding site stabilises binding of the DIII-DIV linker to the inactivation receptor site, strengthening the conclusions of the paper.

We thank the reviewer for pointing out that there do exist parameters for different PIP sub-species and have corrected our statement on page 14 line 16 to reflect this. We have not run additional CG simulations with each of these parameters but use the all-atom simulations to examine the interactions of phosphates at specific positions.

In our atomistic simulations, we backmapped both PI(4,5)P2 and PI(4)P in the binding site to study their specific interactions. We chose to focus on PI(4,5)P2 given its physiological significance. However, we agree that differences in binding with PI(3,4)P2 would be interesting and warrants future investigation. We also note that the newer Martini3 forcefield would be useful in further work to differentiate between PIP subspecies interactions.

Detailed Comments

We thank the reviewers for their thorough reading and helpful comments which has allowed us to further strengthen the manuscript. Below, we provide point-by-point responses to their suggestions.

**Reviewer #1 (Recommendations For The Authors):**

I don't have many suggestions for the manuscript, just a few text edits. Of course, experimental analysis would bolster the claims made in the text, but I don't believe that this is necessary, given the quality of the data.

I understand the focus on the PIP lipids, but it's a shame that the high binding likelihood of glycosphingolipid isn't considered or analysed in any way. This is an especially interesting lipid from the point-of-view of raftlike membrane domains. Given the potential role of raft-like domains in sodium channel function, I feel this would be worth a paragraph or two in the discussion.

We thank the reviewer for bringing our attention to this interesting point. Glycolipids accumulate around Nav1.4 in our complex membrane simulations, however, given reports that carbohydrates tend to interact too strongly in the Martini2.2 forcefield (Grünewald et al. 2022, Schmalhorst et al. 2017) and there are no specific residues on Nav1.4 that interact preferentially with glycolipid species, we chose not to focus on this. However, we have noted that interactions with other lipids deserve further attention in our revised discussion.

The analyses have been run using Martini 2. I don't suggest the authors repeat using the Martini 3 force field, but some mention of this in the discussion would be good.

We have added the following statement to the discussion: “Our coarse grain simulations were carried out using the Martini2.2 forcefield, for which lipid parameters for many plasma membrane lipids have been developed. We expect that future investigations of lipid-protein interactions will benefit from use of the newer, refined Martini 3 forcefield (Souza et al. 2021) as parameters become available for more lipid types.

This might just be an oversight, but no mention is made of an elastic network applied to the backbone beads.

Lack of a network has been known to cause the protein to collapse, so if this is missing, I'd like to see an RMSD to show that the protein dynamics are not compromised.

While no elastic network was used in our original CG simulations, weak protein backbone restraints (10 kJ mol-1 nm-2) used in our simulations allowed us to maintain the structure while allowing some protein movement. However, following the suggestion of reviewer 3, we conducted additional simulations with an elastic instead of backbone restraints as described in the results on page 9 line 30-37 (and in Fig 4E) of the revised manuscript.

Minor

•In Fig 3B, are these lipids binding to the channel at the same time? And therefore do the authors see cooperativity?

The Fig 3B caption has been amended in the revised manuscript to read “Representative snapshots from the five longest binding events from different replicates, showing the three different PIP species (PIP1 in blue, PIP2 in purple and PIP3 in pink) binding to VSD-IV and the DIII-IV linker.” We cannot comment on PIP cooperativity based on these simulations shown in Fig 3, due to the artificially high concentrations used here; however, in model complex membrane simulations we see co-binding of PIPs at the binding site. This is likely due to PIP’s ability to accumulate together and the high density of positively charged residues in the region, attracting and supporting multiple PIP bindings.

•What charges were used for the atomistic PIP lipids? Does this match the CG lipids?

We used the CHARMM-GUI PIP parameters for the atomistic simulations. SAPI24 (PIP2) has a headgroup charge of –4e which is one less negative charge than the CG PIP2; whereas SAPI14 (PIP1) has a charge of –3e which is the same as the CG PIP1. We have explicitly included this charge information in the updated Methods of the manuscript (on page 15-16).

•Line 259-260: "we performed embedded three structures"

Corrected in the revised manuscript.

•Line 272: "us" should be "µs"

Corrected in the revised manuscript.

•Line 434: kJ/mol should probably also have 'nm-2' included

Corrected in the revised manuscript.

•What charge state titratable residues were set to, and were pKa analyses done to decide this?

Charge states were assigned to default values at neutral pH. We appreciate that future studies could examine this more carefully using constant pH simulations or similar.

•It's stated that anisotropic scaling is used the AT sims - is this correct? If so, is there a reason this was chosen over semi-isotropic scaling?

Anisotropic scaling was used for the atomistic simulations allowing all box dimensions to change independently.

•I would recommend in-house analysis scripts are made available on GitHub or similar, just so the details can be seen.

Per the reviewer’s request, the Jupyter notebooks used for the following analysis has been made available on GitHub (https://github.com/etaoster/etaoster.github.io/tree/main/nav_pip_project ).

- Lipid DE
- Contact occupancy + outlier plots
- Binding duration plots
- Minimum distance plots
- Number of ARG/LYS plots
- PIP Occupancy, binding duration, gating charge residues
- RMSD, RMSF and distance between IFM and its binding pocket (using MDAnalysis)
- Atomistic PIP headgroup interaction analyses and plots (using ProLIF)

As a final note, I am *NOT* saying this needs to be done for the current study, but I recommend the authors try the PyLipID package (https://github.com/wlsong/PyLipID) if they haven't yet, as it might be useful for similar projects they run in the future (i.e. for binding site identification, accurate binding kinetics calculations, lipid pose generation etc.).

We thank the reviewer for this suggestion and will keep this in mind for future projects.

**Reviewer #2 (Recommendations For The Authors):**

Lin Y., Tao E., et al. used multiscale MD simulations to show that PI(4,5)P2 binds stably to an inactivated state of Nav channels at a conserved site within the DIV S4-S5 linker, which couples the voltage sensing domain (VSD) to the pore. The authors hypothesized that PI(4,5)P2 prolongs inactivation by binding to the same site where the C-terminal tail is proposed to bind during recovery from inactivation. They convincingly showed that PI(4,5)P2 reduces the mobility of both the DIV S4-S5 linker and the DIII-IV linker, thus slowing the conformational changes required for the channel to recover to the resting state. They also conducted MD simulations to show that phosphoinositides bind to VSD gating charges in the resting state of Nav channels. These interactions may anchor VDS at the resting state and impede its activation. Their results provide a mechanism by which phosphoinositides alter the voltage dependence of activation and the recovery rate from inactivation, an important step for developing novel therapies to treat Nav-related diseases. However, the study is incomplete lacks the expected confirmatory studies which are relevant to such proposals.

The authors identified a novel binding between phosphoinositides and the VSD of Nav and showed that the strength of this interaction is state-dependent. Based on their work, the affinity of PIPs to the inactivated state is higher than the resting state. This work will help pave the way for designing novel therapeutics that may help relieve pain or treat diseases like arrhythmia, which may result from a leftward shift of the channel's activation. However, the study lacks the expected confirmatory studies which are relevant to such proposals. For example, one would expect that the authors would mutate the positive residues that they claim to make interactions with phosphoinositides to show that there are much fewer interactions once they make these mutations. Another point is that the authors found that the main interaction site of PIPs with Nav1.4 is the VSD-DIV and DIII-DIV linker, an interaction that is expected to delay fast inactivation if it happens at the resting state. The authors should make a resting state model of the Nav1.4 channel to explain the recent experimental data showing that PIP2 delays the activation of Nav1.4, with almost no effect on the voltage dependence of fast inactivation.

Major concern:

(1) Lack of confirmatory experiments, e.g., mutating the positive residues that show a high affinity towards PIPs to a neutral and negative residue and assessing the effect of mutagenesis on binding.

(2) Nav1.4 is the only channel that has been studied in terms of the effect of PIPs on it, therefore the authors should build a resting state model of Nav1.4 and study the effect of PIPs on it. Minor points:

Following the reviewer’s suggestion we have conducted new simulations demonstrating that there are notably fewer protein-PIP interactions after performing charge neutralizing and charge reversal mutations to the positive residues as shown in the new Fig S6.

The reviewer mentions that if PIPs interact with the VSD-DIV and DIII-DIV linker in the resting state that it could delay fast inactivation. However as described in the original manuscript and depicted in the schematic (Fig 7) the C-terminal domain impeded PIP binding at the position in the resting state (but not the inactivated state), meaning that PIP does not bind in the resting state to delay fast inactivation. We have clarified this statement in the text on page 14 lines 1-2.

Following the reviewers suggestion we have examined PIP binding to a model of the resting state of Nav1.4 (in addition to the resting state of Nav1.7 described in the original manuscript) as described on page 12 lines 28-30 (and in Fig S12). Similar to what we saw for Nav1.7 PIP binding to VSDI-III can impair activation of the channel.

There are a lot of wrong statements in many areas, e.g., "These diseases 335 are associated with accelerated rates of channel recovery from inactivation, consistent with our observations that an interaction between PI(4,5)P2 and the residue corresponding to R1469 in other Nav 337 subtypes could be important for prolonging the fast-inactivated state." Prolonging the fast inactivated state would actually reduce recovery from inactivation and not accelerate it.

We disagree with this statement from the reviewer which may have come from a misreading of the mentioned sentence. Our statement in the original manuscript is consistent with the the original experiments that show that the presence of PIP prolongs the time spent in the fast inactivated state. Mutations at the PIP binding site are likely to reduce PIP binding, and with less PIP present the channel will recover from inactivation more quickly. We have reworded this sentence for clarity on page 13 line 27-30.

**Reviewer #3 (Recommendations For The Authors):**

As mentioned in the public review, overall, I am impressed with the manuscript and do think the conclusions are supported. There are, however, quite a few mistakes, mostly minor (listed below). Additionally, I do have a few questions and several extensions that could be done and I mention a few but fully realize many of those could be outside of the scope of the current manuscript.

We greatly appreciate the time taken by Reviewer 3 to carefully review our manuscript and provide detailed comments. We believe their suggestions have helped to improve our manuscript.

First comments are in general about the PIP subtype.

- In the paper you claim:

L196, "However, this loss of resolution prevents distinction between phosphate positions on the inositol group and does not permit analysis of protein conformational changes induced by PIP binding"

L367, "it does not distinguish between phosphate positions within each charge state (e.g. PI(3,4)P2 vs PI(4,5)P2)."

This is not true the PIP2 most commonly used in Martini 2 is from dx.doi.org/10.1021/ct3009655 and is a PI(3,4)P2 subtype. Also other extensions and alternative parameters exist for PIPs in Martini 2 e.g.http://cgmartini.nl/index.php/tools2/other-tools - Martini lipid .itp generator has all three main variants of both PIP1 and PIP2.

As described in the response to the public review we are grateful for the reviewer for pointing out that there do exist parameters for different PIP sub-species and have corrected our statement on page 14 to reflect this, and clarified the parameters chosen in the methods section (page 16 line 2-3). We have not run additional CG simulations with each of these parameters in the current work but use the all-atom simulations to examine the interactions of phosphates at specific positions.

- One detail that is missing in the manuscript is some mention of the charge state of the PIPs e.g. Fig.1D does not specify and Fig.4D PIP2 looks like -2 on position 5 and -1 on position 4. Which I think fits the used SAPI24, please specify. Also, what if you use SAPI25 with the flipped charges would that significantly alter the results?

The charge state of PIP2 is -2e on the 5’ phosphate and -1e on the 4’ phosphate, using the SAPI24 CHARMM lipid parameters. We have ensured that this charge information is stated clearly in the revised manuscript in the methods section on page 16 (line 21). We considered looking at SAPI25, however we expected that it would behave quite similarly, given that the PIP headgroup can adopt slightly different poses and orientations within the binding site across replicates and does fluctuate over simulations (Fig S8). We have noted this in the revised discussion on page 14 line 15-17.

- I was very intrigued and puzzled by the lower binding of PIP3 vs PIP2 in the Martini simulations. Could it be that PIP3 has a harder time fully entering the binding site, or maybe just sampling? i.e. and its lower number of binding events is a sampling issue.

We agree with the reviewer that PIP3 is less able to access the binding site than PIP2, likely because of its larger size. This might also be why we see PIP1 binding at the location via a more buried route (since it has the smallest headgroup size). However, PIP1 does not have enough negative charge to keep it in the binding site. It seems to be a Goldilocks-like situation where PIP2 has the optimal size and charge to allow access and stable binding at the site. We also see that when PIP3 enters the binding site it leaves before the end of the simulations. While it is hard to prove statistical significance given the number of binding and dissociation events even with the high and equal concentrations of all three PIP species in the enriched PIP membrane CG simulations, the data strongly suggests preferential binding of PIP2 over PIP3.

Also the same L196 sentence as above "However, this loss of resolution prevents distinction between phosphate positions on the inositol group and does not permit analysis of protein conformational changes induced by PIP binding". The later part is also wrong, there are no conformational changes due to the restraints on the protein backbone, from methods "backbone beads were weakly restrained to their starting coordinates using a force constant of 10 kJ mol−1nm−2". Martini in general might have a hard time with some conformational changes and definitely cannot sample changes in secondary structure, but conformational changes can, and have on many occasions, been successfully sampled (even full ion channel opening and closing).

On a similar note, in L179 you mention "owing to the flexibility of the linker." Hose does this fit with simulation with position restraints on all backbone atoms?

We applied fairly weak restraints to the backbone only – therefore we still observe some flexibility in the highly flexible loop portion of the linker, where sidechains are able to flip between membrane-facing and cytosol-facing orientations.

However, after reading the comments from the reviewer we have run additional simulations with an elastic network rather than backbone restraints on the DIII-DIV linker which have given further insight. As seen in Fig 4E and described in the results paragraph on page 9 line 30-37 of the revised manuscript, we can see that the presence of PIP does stabilise the linker in its receptor site. To accentuate this effect, we also ran simulation of the ‘IQM’ mutant known to have a less stable fast inactivated state due to weaker binding to the receptor. Without backbone restraints we can see partial dissociation of the DIII-DIV linker from the receptor that is partially rescued by the presence of PIP.

I know the paper focuses on PIPs, also very nicely in Fig.2B and Fig. S1-2 the lipid enrichment is shown for other lipids, but why show all lipid classes except cholesterol? And, for the left-hand panels in Fig. S1-2 those really should be leaflet specific - as both the membrane and protein are asymmetric.

The depletion/enrichment of Cholesterol is shown in Fig 2B and as are the Lipid Z-Density maps and contact occupancy structures a (in row 5 of Fig S2, labeled as CL in yellow). The Z-density maps are meant to provide an overall summary of lipid distribution. The contact occupancy structures showing the transverse views and intracellular/ extracellular views provide a better indication of the occupancy across the different leaflets.

In L237 for the comparison of Cav2.2 and Kv7.1 bound to PI(4,5)P2 structures: They do agree well with the PIP1 simulations but not as much for the main PIP2 binding site. If you look in the CG simulations, is there another (not the main) PIP2 binding site at that same location (which might also be stable in AA simulations)?

In some replicates of the CG simulations, we identify stable PIP1 binding via the other orientation (i.e. the one that overlaps with the Cav2.2 and Kv7.1 structures). Since we did not directly observe any PIP2 binding events from the other orientation, we did not run any backmapped atomistic simulations with PIP2 at this position. However, the binding site residues that the PIP1/2 headgroup binds to are the same regardless of which side PIP1/2 approaches from. We would expect that PIP2 bound from the alterative position is also stable.

Two references I want to put for consideration to the authors, for potential inclusion if the authors find their inclusion would strengthen the manuscript. This one gives a good demonstration of using the same PM mixture to define lipid protein fingerprints with Martini:

https://pubs.acs.org/doi/10.1021/acscentsci.8b00143.

And this one https://pubmed.ncbi.nlm.nih.gov/33836525/ shows how Nav1.4 function could also be affected by general changes in bilayer properties (in addition to the specific lipid interactions explored here).

We thank the reviewer for bringing to our attention these two relevant references that will help to respectively substantiate the use Martini to study membrane protein-lipid interactions, as well as, why Nav channels are interesting to study in the context of their membrane environment (and also the potential implications with drugs that can bind from within the membrane). We have added these citations to the introduction and discussion.

Minor comments and fixes:

L2, Title: A binding site for phosphoinositide modulation of voltage-gated sodium channels described by multiscale simulations

The title reads very strangely to me, should it be "A binding site for phosphoinositide" ; "modulation".We thank the reviewer for this comment - title has been updated to: A binding site for phosphoinositides described by multiscale simulations explains their modulation of voltage gated sodium channels.

L25, Abstract, "The phosphoinositide PI(4,5)P2 decreases Nav1.4 activity by increasing the difficulty of channel opening, accelerating fast activation and slowing recovery from fast inactivation." Assuming this is referring to results from Gada et al JGP, 2023 should this not be "accelerating fast inactivation"?

Corrected in the revised manuscript.

L71 maybe good to write the longer version of IFM on first use e.g. Ile-Phe-Met (IFM), as to not mistake it for some random three letter acronym.

Corrected in the revised manuscript.

L109, Fig.2. Maybe change the upper and lower leaflet to intracellular and cytoplasmic leaflets (or outer / inner). In D "(D) Distribution of PIP binding occupancies (left)" something missing can I assume, for/over all lipids exposed residues. Also, for D I am a little confused how occupancy is defined as the total occupancy per residue dose not add up to 100.

The figure has been updated with intracellular and cytoplasmic leaflet labels. The binding occupancy distribution boxplot shows binding occupancies for all lipid exposed residues. In our analysis, we define contact occupancy as the proportion of simulation time in which a lipid type is within 0.7 nm of a given residue. It is possible for more than one lipid to be within this cut in any given frame – that is, both a PIP and PE can be simultaneously bound.

L160 "occurring the identified site" in the

Corrected in the revised manuscript.

L170 "PIP3 (headgroup charge: -7e) has interacts similarly to PIP1," - remove hasCorrected in the revised manuscript.

L194, "reducing system size" the size does not change, I am assuming you want to say reducing the numberof particles?

Corrected in the revised manuscript.

L252, Fig.6 "(B) Occupancy of all PIPs (PIP1, PIP2, PIP3) at binding site residues in the three systems" A little confusing, initially was expecting 3x3 data points per residue, maybe change to, Combined occupancy of all PIPs...

Corrected in the revised manuscript.

L253, Fig.6 D, I don't really have a good suggestion for improvement here, so this is just a FYI that this panel was very confusing for me and took some time to figure out what is shown.

We have added to the caption of Fig. 6D to try to clarify this panel.

L257, Fig.6 (F) not in bold

Corrected in the revised manuscript.

L259 "PIP binding, we performed embedded three structures of Nav1.7" something missing?

Corrected in the revised manuscript.

L272, "In triplicate 50 us coarse-grained simulations" us instead of (micro_greek)s

Corrected in the revised manuscript.

L272, that paragraph how long/many simulations only reported for the inactivated Nav1.7 system not the Nav1.7-NavPas chimera, which I am assuming is the same?

Corrected in the revised manuscript.

L297, "marked by both shortened inactivation times", can I assume this is: shortened times to inactivation (i.e. to get inactivated not times in the inactivated states)?

Corrected in the revised manuscript.

L331, "are conserved in Nav1.1-1.9 (Fig. 5D)," Fig.5CCorrected in the revised manuscript.

L353, "channel opening []" [] maybe a missing reference?

Thank you for pointing out this oversight - Goldschen-Ohm et al. has been cited here.

L394, "The composition of the complex mammalian membrane is as reported in Ingólfsson, et al. (38)." Ref 38 is the "Computational lipidomics of the neuronal plasma membrane" which indeed uses the 63 component PM but the original reference for the average 63 lipid mixture PM is dx.doi.org/10.1021/ja507832e.

Corrected in the revised manuscript.

L404, "Additionally, a model Nav1.7 with all four VSDs in the deactivated state using Modeller (40)." Something missing, e.g. was also built and simulated for ...

Corrected in the revised manuscript.

Table S1 "Disease information", I am guessing this should be Disease information; mechanism?Of the x5 entries two have mechanism, one has "; unknown significance ", one has "; unknown" maybe clarify in title and make same if unknown.

Corrected in the revised manuscript.

Table S1 and S2 have different styles.

The tables have been amended to have the same style.

Fig. S3 "for all 12 lipid types in the mammalian membrane " there are many more lipid types in a typical PM (hundreds) and 63 in the PM mixture simulated here, so maybe write: 12 lipid classes?

Corrected in the revised manuscript.

Fig.S6 PIP headgroup, can I assume that is for the bound PIP only, please specify.

Only a single PIP at the identified binding site was backmapped into all cases of atomistic simulations. We have now clarified this point in the methods, results and the FigS6 caption.

Writing of PI(4,5)P2 and PI(4)P1 most of the time use 1 and 2 as subscripts but not always (at least not in SI), also the same with Nav vs Na_v (v subscript) and even NAV (in Table S1).

Subscripts have been implemented in the updated Supplementary Information (as well as within various figures and throughout the manuscript).
